# A Screen of *Coxiella burnetii* Mutants Reveals Important Roles for Dot/Icm Effectors and Host Autophagy in Vacuole Biogenesis

**DOI:** 10.1371/journal.ppat.1004286

**Published:** 2014-07-31

**Authors:** Hayley J. Newton, Lara J. Kohler, Justin A. McDonough, Morayma Temoche-Diaz, Emerson Crabill, Elizabeth L. Hartland, Craig R. Roy

**Affiliations:** 1 Department of Microbial Pathogenesis, Yale University School of Medicine, New Haven, Connecticut, United States of America; 2 Department of Microbiology and Immunology, University of Melbourne, Parkville, Victoria, Australia; Duke University, United States of America

## Abstract

*Coxiella burnetii* is an intracellular pathogen that replicates in a lysosome-derived vacuole. The molecular mechanisms used by this bacterium to create a pathogen-occupied vacuole remain largely unknown. Here, we conducted a visual screen on an arrayed library of *C. burnetii* NMII transposon insertion mutants to identify genes required for biogenesis of a mature *Coxiella*-containing vacuole (CCV). Mutants defective in Dot/Icm secretion system function or the PmrAB regulatory system were incapable of intracellular replication. Several mutants with intracellular growth defects were found to have insertions in genes encoding effector proteins translocated into host cells by the Dot/Icm system. These included mutants deficient in the effector proteins Cig57, CoxCC8 and Cbu1754. Mutants that had transposon insertions in genes important in central metabolism or encoding tRNA modification enzymes were identified based on the appearance filamentous bacteria intracellularly. Lastly, mutants that displayed a multi-vacuolar phenotype were identified. All of these mutants had a transposon insertion in the gene encoding the effector protein Cig2. Whereas vacuoles containing wild type *C. burnetii* displayed robust accumulation of the autophagosome protein LC3, the vacuoles formed by the *cig2* mutant did not contain detectible amounts of LC3. Furthermore, interfering with host autophagy during infection by wild type *C. burnetii* resulted in a multi-vacuolar phenotype similar to that displayed by the *cig2* mutant. Thus, a functional Cig2 protein is important for interactions between the CCV and host autophagosomes and this drives a process that enhances the fusogenic properties of this pathogen-occupied organelle.

## Author Summary

*Coxiella burnetii* is the causative agent of the human disease Q fever. This bacterium uses the Dot/Icm type IV secretion system to deliver effectors into the cytosol of host cells. The Dot/Icm system is required for intracellular replication of *C. burnetii*. To determine the contribution of individual proteins to the establishment of a vacuole that supports *C. burnetii* replication, we conducted a visual screen on a library of *C. burnetii* transposon insertion mutants and identified genes required for distinct stages of intracellular replication. This approach was validated through the identification of intracellular replication mutants that included insertions in most of the *dot* and *icm* genes, and through the identification of individual effector proteins delivered into host cell by the Dot/Icm system that participate in creating a vacuole that supports intracellular replication of *C. burnetii*. Complementation studies showed convincingly that the effector Cig57 was critical for intracellular replication. The effector protein Cig2 was found to play a unique role in promoting homotypic fusion of *C. burnetii* vacuoles. Disrupting host autophagy phenocopied the defect displayed by the *cig2* mutant. Thus, our visual screen has successfully identified effectors required for intracellular replication of *C. burnetii* and indicates that Dot/Icm-dependent subversion of host autophagy promotes homotypic fusion of CCVs.

## Introduction

*Coxiella burnetii* is a highly infectious human pathogen responsible for a global zoonotic disease called Q fever. Inhalation of contaminated aerosols by humans can lead to an acute systemic illness or a more serious chronic infection that commonly presents as endocarditis [1], [2]. The animal reservoirs for *C. burnetii* include domesticated livestock, and transmission to humans from these animals can lead to outbreaks of disease, such as the Q-fever epidemic that was linked to dairy goat farms in the Netherlands [2].

Phase I strains of *C. burnetii* produce a lipopolysaccharide molecule that has a complex O-antigen polysaccharide chain that protects the bacteria from being killed by host serum [3]. Phase II variants of *C. burnetii* produce a truncated O-antigen polysaccharide and can be isolated from both infected animals and bacteria cultured *ex vivo* [3], [4]. Although most strains of *C. burnetii* exhibit phase variation and switch between phase I and phase II spontaneously, a phase II variant of the *C. burnetii* Nine Mile strain RSA493 called clone 4 (NMII) is phase locked because it has a chromosomal deletion that eliminates several genes required for the synthesis of O-antigen polysaccharide, which makes this strain incapable of causing systemic disease in guinea pig and mouse models of infection and enhances innate immune detection [3], [4]. Nonetheless, it has been shown that the NMII strain is indistinguishable from the isogenic phase I strain (NMI) in tissue culture models of infection that measure the ability of *C. burnetii* to replicate in human cells, which include studies in primary human monocyte-derived macrophages [5], [6]. Importantly, NMI and NMII encode the same array of virulence determinants that have evolved for manipulating cellular functions important for intracellular replication.

Intracellular replication of *C. burnetii* requires formation of a specialized membrane-bound compartment termed the *Coxiella*-containing vacuole (CCV). After cells internalize *C. burnetii* there is host-directed transport of the CCV through the endocytic pathway, which delivers the bacteria to the low pH environment of a lysosome [7], [8]. Intracellular *C. burnetii* resist the hydrolytic and bactericidal activities inside the lysosome and the acidic pH of this organelle is required to stimulate *C. burnetii* metabolism, which enables the bacteria to survive and replicate intracellularly [9], [10]. Although the molecular mechanisms used by *C. burnetii* to transform a lysosome into a replication-permissive compartment remain unclear, there is evidence that this compartment interacts with vesicles derived from the host autophagic and secretory pathways [11]–[13]. This results in a compartment containing *C. burnetii* that displays the host autophagy proteins LC3 and Rab24 [12], and late endosomal/lysosomal proteins such as LAMP1, cathepsin D and the vacuolar type H+ ATPase [14]. It has been shown recently that the CCV accumulates host cholesterol resulting in robust localization of lipid raft proteins flotilin 1 and 2 and that this vacuole is encompassed by an F-actin cage [15], [16]. Thus, the CCV is a unique pathogen-occupied organelle that is generated upon fusion with host lysosomes.

Another unique feature of the CCV is that it has the ability to fuse promiscuously with other endosomal compartments in the cell, which consumes cellular lysosomes and results in the formation of a large lysosome-derived compartment in the infected cell [17], [18]. Co-infection studies have shown that the ability of the CCV to fuse with other endocytic compartments will promote fusion of pre-existing phagolysosomes containing inert latex-bead particles with the CCV and will also promote the fusion of vacuoles containing other pathogenic microbes with the CCV [19], [20]. Importantly, if a cell is independently infected with multiple *C. burnetii*, the ability of the bacteria to stimulate homotypic fusion of lysosome-derived compartments will lead to the formation of a single CCV in the infected cell [18]. Inhibition of bacterial protein synthesis after infection prevents bacterial manipulation of endosomal dynamics and results in contraction of the spacious CCV to create a tight-fitting membrane that surrounds bacteria residing in the CCV lumen [18]. In addition to manipulating the host membrane transport and fusion pathways to produce a mature CCV, *C. burnetii* also promotes host viability by actively preventing apoptosis [21], [22]. Manipulation of membrane transport and inhibition of apoptosis are both predicted to be pathogen-directed strategies that enable *C. burnetii* to replicate efficiently in mammalian host cells.

Deciphering the unique molecular mechanisms that *C. burnetii* uses to manipulate the host has become possible with the development of axenic culture conditions. Formerly classified as an obligate intracellular bacterium, it has been shown that *C. burnetii* replicates axenically in Acidified Cysteine Citrate Media (ACCM) with 5% CO_2_ and low oxygen conditions [23], [24]. Subsequently, genetic approaches were developed to isolate transposon-insertion mutants and mutants with targeted gene deletions [25]–[27], which were used to demonstrate that the Dot/Icm type IVB secretion system encoded by *C. burnetii* is essential for intracellular replication [27]–[29]. This secretion system is genetically and functionally related to the Dot/Icm system of the human pathogen *Legionella pneumophila* [30]–[32]. In *L. pneumophila*, the Dot/Icm system facilitates intracellular replication by translocating into the host cytosol approximately 300 different effector proteins [33]. These effectors rapidly modulate the host cell biology to direct the remodeling of the *Legionella*-containing vacuole (LCV), which prevents fusion with lysosomes and promotes fusion of secretory vesicles to create a vacuole that supports intracellular replication [33]. The biochemical function of a small proportion of these effectors has been elucidated but correlating these functions to pathogenesis is hampered by a large degree of functional redundancy both in terms of multiple paralogs with mirroring functions [34] and dissimilar effectors targeting the same host cell pathways [35], [36]. With few exceptions, deletion of a gene encoding a *L. pneumophila* effector does not typically have a measurable impact on the ability of the bacterium to replicate intracellularly. It is thought that the diversity of the natural protozoan hosts *L. pneumophila* encounters in nature has resulted in the selection of functionally-redundant effectors that mediate survival in specific protozoan hosts [36].

The *L. pneumophila* Dot/Icm system initiates effector translocation immediately upon contact with a host to prevent the LCV from engaging the host endocytic pathway [37], [38]. By contrast, the *C. burnetii* Dot/Icm system does not translocate effectors until the bacteria are delivered to lysosomes and become metabolically active in an acidified vacuole [39]. Given their divergent intracellular infection strategies it is predicted that there will be minimal overlap in the function of the effectors encoded by *L. pneumophila* when compared to *C. burnetii*, which is supported by the observation that few *bone fide* homologs of *L. pneumophila* effectors are encoded in the *C. burnetii* genome. To date, over 100 *C. burnetii* Dot/Icm effectors have been identified using a range of methods [28], [40]–[46]. The translocation of the majority of these effectors was observed using *L. pneumophila* as a surrogate effector delivery platform. Several *C. burnetii* effectors have been implicated in preventing apoptosis [43], [47], including the ankyrin repeat-containing protein AnkG that infers an anti-apoptotic phenotype on *L. pneumophila* [43]. It is predicted that *C. burnetii* effectors will also function to control membrane traffic, as demonstrated by the effector CvpA interacting with clathrin-coated vesicles [48], and to manipulate other aspects of the host biology important for intracellular replication. Because *C. burnetii* replicates exclusively inside mammalian hosts it is predicted that there will be less functional redundancy in the cohort of *C. burnetii* effector proteins compared to what is observed for *L. pneumophila* effector proteins, and that loss of single effectors may impact CCV formation. This means that it should be possible to identify effectors important for intracellular replication, and that determining the function of these effectors will increase our understanding of CCV development.

Here, we conducted a visual screen on an arrayed library of random transposon insertion mutants of *C. burnetii* NMII to identify genes important for formation of the mature CCV. This approach was successful and resulted in the identification of genes that are important for the intracellular lifestyle of *C. burnetii*. The requirement for a functional Dot/Icm system in biogenesis of the CCV was evident. Insertions that inactivated genes encoding structural components of this secretion apparatus were identified in addition to insertions in genes encoding regulatory factors that govern expression of the Dot/Icm system. Importantly, these studies have identified several effector proteins that play important and distinct roles during intracellular replication. Specifically, using this approach we reveal a genetic interaction between the effector Cig2 and the host autophagy pathway. These data indicate that Cig2 function is required for robust interactions between the CCV and host autophagosomes, and that this maintains the CCV in an autolysosomal stage of maturation.

## Results

### Construction of an arrayed library of *C. burnetii* transposon mutants

The plasmid pKM225 encoding a Himar1 TnA7 transposase was used to introduce a transposon encoding chloramphenicol resistance and a mCHERRY fluorescent protein randomly onto the genome of the *C. burnetii* NMII strain RSA493. The mutagenesis procedure was optimized to reduce isolation of siblings containing identical transposon insertions and to reduce the number of spontaneous mutants defective for Dot/Icm function (described in Materials and Methods). After optimization, 3,840 transposon insertion mutants were isolated as single clones from 40-independent pools. These clones were then arrayed and expanded in 96-well plates containing ACCM-2. We were successful in expanding 84.6% of the clones (3,237 mutants) in liquid ACCM-2 under antibiotic selection. To determine the degree to which these clones represented independent mutants with different sites of transposon insertion, we determined the site of insertion for a total of 459 mutants using a two-stage semi-degenerate PCR amplification and sequencing protocol. This analysis confirmed that isolated clones had single transposon insertions distributed randomly across the *C. burnetii* genome. Additionally, this analysis revealed several mutants that had transposon insertions in genes encoding known effectors of the Dot/Icm system (Tables S1, S2, S3, S4, S5).

### A visual screen identifies *C. burnetii* mutants with vacuole biogenesis defects

**Figure 1:**
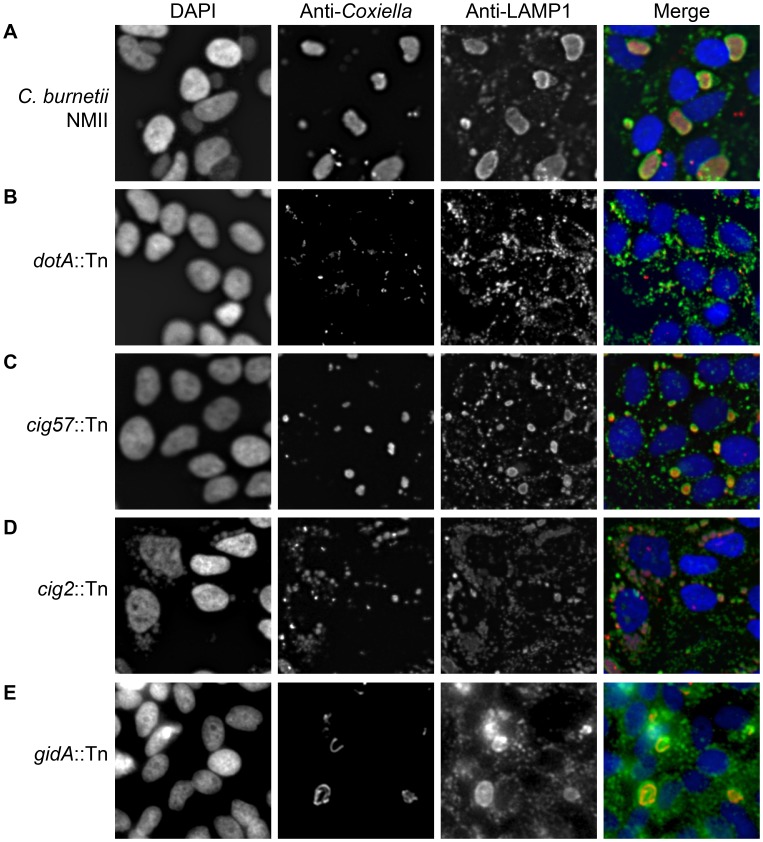
Transposon mutants of *C. burnetii* display different intracellular phenotypes. Transposon mutants were subject to a vacuole formation assay in which 96-well plates of HeLa 229 cells were infected, at an MOI of approximately 500, with individual transposon mutants. Following a 96 h infection period, the infection was fixed and stained with anti-*Coxiella* (red), anti-LAMP1 (green) and Hoechst dye (blue) and observed with low magnification fluorescence microscopy. (A) The parental strain *C. burnetii* NMII displayed a large CCV in the majority of HeLa cells. (B) A cohort of mutants showed no intracellular replication as demonstrated by *dotA*::Tn, (C) another category produced smaller replicative vacuoles such as that seen with *cig57*::Tn, (D) transposon insertions that disrupted *cig2* resulted in the appearance of multiple vacuoles in a single cell, and (E) a small proportion of mutants, such as *gidA*::Tn, displayed CCVs with an abnormal shape due to filamentous replication of the *C. burnetii*.

The arrayed library of *C. burnetii* NMII transposon mutants was analyzed using a visual assay that assessed the ability of individual mutants to form vacuoles that support intracellular replication. Specifically, HeLa 224 cells distributed in 96-well glass-bottom plates were infected with individual mutants at an MOI of approximately 500 and then the cells were incubated for 96 h. Cells were fixed and stained with antibodies specific for *Coxiella* (red) and LAMP1 (green), and the DNA was labeled with Hoechst dye (blue). The ability of each mutant to form a vacuole that supported intracellular replication was assessed by direct examination using fluorescence microscopy. Importantly, the parental NMII control strain and most of the *C. burnetii* transposon insertion mutants formed a single spacious vacuole filled with replicating bacteria (Figure 1A). Additionally, of the 459 mutants for which the transposon insertion site was determined, we found that 324 mutants (71%) did not display a discernable vacuole formation defect in this visual assay, which included several mutants having insertions in genes encoding known effectors of the Dot/Icm system (Table S5). Lastly, as described in detail below, many of the mutants that displayed vacuole formation defects had insertions in genes that would be predicted to affect intracellular replication. Thus, confidence was high that this screen would identify a unique cohort of genes important for *C. burnetii* replication in mammalian cells.

There were four distinct mutant phenotypes revealed in this visual screen (Figure 1). A severe defect characterized as no detectible intracellular replication in the visual screen was observed for 74 mutants having transposon insertions that were distributed among 21 different protein-coding regions and six different intergenic regions (Table S1). At 96 h post-infection these mutants were observed as single bacteria inside of LAMP1-positive vacuoles (Figure 1B). Forty-two transposon mutants displayed a reduced ability to replicate intracellularly as determined by their presence in small vacuoles containing fewer bacteria compared to vacuoles containing the control strain (Figure 1C, Table S2). Nine transposon mutants displayed filamentous growth inside of host cells (Figure 1E, Table S3), suggesting that these bacteria were under stress or defective for cell division. Lastly, there were 10 transposon mutants isolated independently that displayed a multi-vacuolar phenotype, which was characterized by the appearance of infected host cells that contained multiple vacuoles each supporting replication of *C. burnetii* (Figure 1D, Table S4). Importantly, every mutant we identified that displayed this multi-vacuolar phenotype had a transposon insertion in the 2,430 bp region encoding the protein Cig2 (Cbu0021).

### Genes essential for Dot/Icm function are highly represented among the mutants defective for intracellular replication

**Figure 2:**
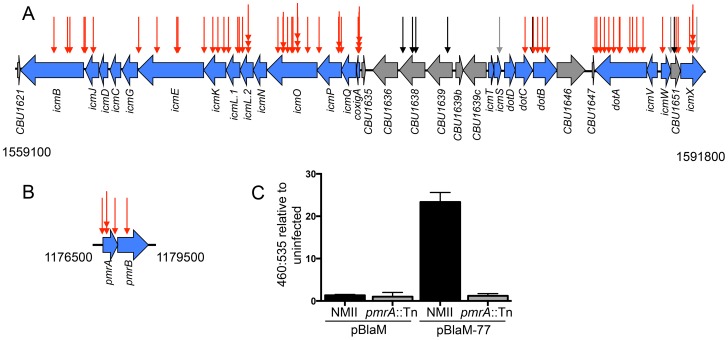
The Dot/Icm system and the PmrAB system are essential for intracellular replication and delivery of *C. burnetii* effector proteins. (A) Indicated are the locations of transposon insertions in the chromosomal regions encoding the Dot/Icm system from 1559100 bp to 1591800 bp, and (B) the PmrAB two-component regulatory system from 1176500 to 1179500 bp. The site of each transposon insertion is represented by an arrow. The mutants that could not replicate intracellularly were assigned red arrows, mutants that produced normal CCVs were assigned black arrows, and mutants that formed small vacuoles were assigned grey arrows. (C) The plasmids pBlaM and pBlaM-77 were introduced into *C burnetii* NMII (black bars) and *pmrA*::Tn (grey bars) to determine whether the PmrAB system was essential for effector translocation. Cleavage of the fluorescent β-lactamase substrate CC4F-AM was determined by calculating the ratio of fluorescence at 460 nm to 535 nm relative to uninfected cells. The graph shows the mean ± SD calculated for three independent samples.

Mutants with transposon insertions in the genes *icmL.2* or *icmD* and mutants with targeted deletions of the genes *dotA* or *dotB* were shown previously to be defective for intracellular replication [27]–[29], which established the essential role the Dot/Icm system has in *C. burnetii* pathogenesis. Here, we identified 66 different intracellular growth mutants harboring a transposon insertion in *dot* and *icm* loci and three mutants that were severely attenuated for intracellular replication with insertions in this region (Figure 2A). The observation that many of the intracellular growth mutants have insertions in the region encoding the Dot/Icm system, as well as the extensive coverage of this region that was obtained, provides addition evidence that the arrayed mutant library contains a random distribution of transposon insertions. Additionally, this analysis indicates that spontaneous unlinked mutations that affect Dot/Icm function did not occur at a high frequency. This was a concern because if spontaneous *dot* and *icm* mutants were encountered at a high frequency then the mutant library would not be effective at identifying effectors essential for intracellular replication, and most intracellular growth phenotypes would not be complemented *in trans* upon introducing plasmids encoding the wild type allele of the disrupted gene. In the pool of 450 transposon mutants that were sequenced, we identified multiple insertion mutations in the coding region located between the *icmQ* and *icmT* genes (Figure 2A). No defects in CCV biogenesis were observed for these mutants, indicating that the genes in this region are not essential for Dot/Icm function. Also, we determined that the hypothetical protein Cbu1651 encoded in this region was not essential for Dot/Icm function because the mutant 16-E10 had an insertion in the *cbu1651* coding region but did not have a vacuole biogenesis defect. Two mutants with the transposon inserted at the 3′ end of the *cbu1651* gene displayed a vacuole biogenesis defect, however, these insertions are predicted to negatively affect expression of *icmX*, which is a gene essential for Dot/Icm function [49].

Previously, it was demonstrated that the *C. burnetii icmD* gene was required for intracellular replication [29]. Additionally, studies on *L. pneumophila* predict that *icmC, icmN, icmT* and *dotD* should also be important for function of the *C. burnetii* Dot/Icm system [50]–[53], and a recent independent study has shown that *C. burnetii* mutants deficient in *dotD*, *icmC* and *icmN* display intracellular replication defects [54]. Although complementation studies and in-frame deletion analysis was not used to more precisely determine the specific *dot* and *icm* genes that were essential for intracellular replication, the region itself was highly represented among mutants with severe intracellular growth defects, which suggested that it should be possible to identify other important genes required for intracellular replication using this library of transposon mutants.

### The two-component regulatory system PmrAB is required for Dot/Icm effector translocation

The response regulator PmrA of *L. pneumophila* is important for intracellular growth of this pathogen as it controls the expression of genes encoding components of the Dot/Icm system and many effectors [55]. The *C. burnetii cbu1227* gene encodes a PmrA homologue [55], which was initially annotated as QseB [30]. The prediction is that PmrA activity is controlled by the sensor kinase PmrB encoded by an adjacent gene in the operon. At least 68 promoter regions in *C. burnetii* contain a consensus PmrA binding site, which included five promoter regions upstream of operons that encode most of the *dot* and *icm* genes [55]. Thus, the prediction is that expression of most *C. burnetii dot* and *icm* genes will require a functional PmrAB system. Consistent with this hypothesis, among the *C. burnetii* mutants identified that were defective for vacuole biogenesis, we isolated three mutants with a transposon insertion in *pmrA*, one mutant with a transposon insertion in *pmrB*, and one mutant with a transposon insertion in the regulatory region upstream of *pmrA* (Figure 2B). To determine if the Dot/Icm system was operational in mutants defective for PmrAB function we introduced a plasmid encoding a β-lactamase reporter (BlaM) fused to the effector protein Cbu0077 that produces the hybrid protein BlaM-77. The BlaM-77 protein was produced in the *pmrA*::Tn mutant strain and effector protein translocation was assayed during host cell infection (Figure 2C). This assay used the substrate CCF4-AM, which when loaded into cells fluoresces at 535 nm (green) following excitation at 415 nm. However, if BlaM-77 is translocated into the host cytosol during infection, the CCF4-AM molecule will be cleaved resulting in a shift in fluorescence to 460 nm (blue). No BlaM-77 translocation was detected when HeLa cells were assayed 24 h after infection with the *C. burnetii pmrA*::Tn mutant (Figure 2C). Thus, the intracellular growth defect displayed by mutants defective in PmrAB function is likely due to a defect in Dot/Icm-dependent delivery of effector proteins important for vacuole biogenesis.

### The effector protein Cig57 is important for intracellular replication

Many *C. burnetii* NMII transposon mutants had an intracellular replication defect that resulted in a significant reduction in the size of vacuoles and the number of bacteria in each vacuole. Included in this category were three transposon insertions that were predicted to result in partial loss-of-function in the Dot/Icm system. These mutants included transposon insertions in the *icmS* gene encoding a chaperone protein that assists in effector translocation [56], a mutant with an insertion located in the 3′ region of *icmX* that would result in the production of an IcmX protein with a small C-terminal deletion, and a mutant with an insertion in the *cbu1651* gene that likely affects the expression of adjacent *dot* and *icm* genes.

**Figure 3:**
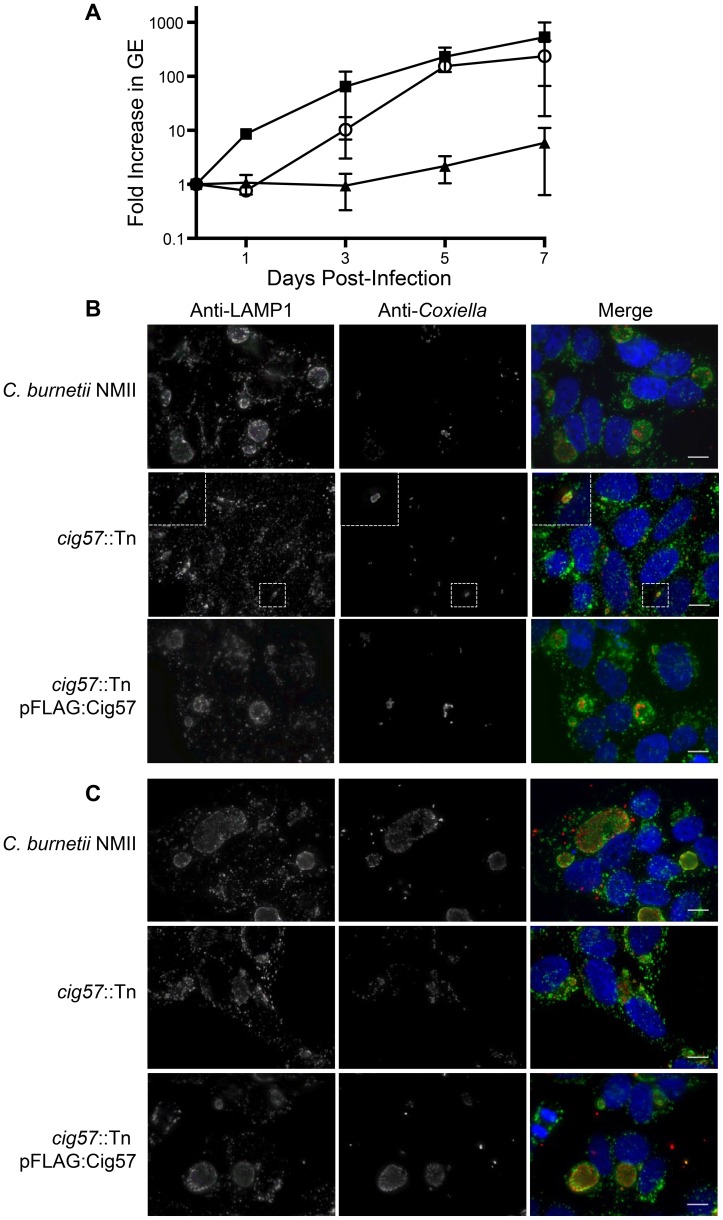
The Dot/Icm effector Cig57 is required for efficient intracellular replication of *C. burnetii*. (A) Intracellular replication of *C. burnetii* NMII (black squares), *cig57*::Tn (black triangles) and *cig57*::Tn pFLAG-Cig57 (open circles). The fold-increase in GEs relative to the inoculum was determined by *dotA*-specific qPCR and is represented here as the mean ± SD of 3 independent infections at days 1, 3, 5 and 7 post-infection. (B,C) Representative micrographs of HeLa cells infected for either 3 days (B) or 5 days (C) with *C. burnetii* NMII, *cig57*::Tn or *cig57*::Tn pFLAG-Cig57. Cell with stained with anti-*Coxiella* antibody (red), anti-LAMP1 antibody (green) and Hoechst dye (blue). Note that the vacuoles containing *cig57*::Tn were smaller and contained few bacteria at each time point compared to the control NMII strain or the complemented *cig57*::Tn pFLAG-Cig57 strain. Scale bars represent 10 µm.

Reduced intracellular replication was also observed in mutants having insertions in genes encoding three different effector proteins, which were Cig57, CoxCC8 and Cbu1754. We isolated 10 intracellular growth mutants having a transposon insertion in *cig57* and the *cig57*::Tn mutant called 3-H3 was analyzed in detail. When intracellular replication was measured over a seven-day period, the 3-H3 strain displayed only a 5-fold increase in genome equivalents (GEs, Figure 3). Introduction of a plasmid-encoded triple FLAG-tagged version of 3×FL-Cig57 (pFLAG:Cig57) into the 3-H3 *cig57*::Tn mutant restored intracellular replication to levels that were equivalent to wild type *C. burnetii*, as determined by 237-fold increase in GEs after a 7-day infection period and the appearance by immunofluorescence microscopy of large spacious vacuoles containing replicating 3-H3 (pFLAG:Cig57) bacteria (Figure 3B & 3C). Importantly, there were no obvious defects observed in the maturation of vacuoles containing 3-H3 as indicated by the presence of both LAMP1 (Figure 3) and cathepsin D (Figure S1) on the vacuoles formed by this mutant. Thus, the effector protein Cig57 likely has a role in modulating host processes important for replication that occur after *C. burnetii* is delivered to a lysosome-derived organelle.

### Screening for intracellular growth mutants identifies a new Dot/Icm effector protein

We identified a strain of *C. burnetii* having a transposon insertion in the gene *cbu1780* and a strain having an insertion in the gene *cbu2072* that had both displayed a severe intracellular growth defect. Both of these genes encode hypothetical proteins, which raised the possibility they might encode novel effectors. To determine if these proteins might encode effectors both Cbu1780 and Cbu2072 were tested for Dot/Icm-dependent translocation using fusion proteins having an amino-terminal BlaM reporter. The resulting BlaM-1780 and BlaM-2072 fusion proteins were produced in *C. burnetii* NM II and the isogenic *icmL*::Tn strain. The BlaM-1780 fusion protein was translocated into the host cytosol when produced in *C. burnetii* with a functional Dot/Icm system, as determined by a significant increase in the 460::535 nm fluorescence ratio to 24.62±2.70. By contrast, no translocation was detected by BlaM-2072, 1.07±0.86. Similarly no translocation was detected for the controls, which included BlaM-1780 and BlaM-2072 produced in the Dot/Icm-deficient mutant and BlaM alone produced in the parental *C. burnetii* NMII strain (Figure S2). Thus, Cbu1780 is an effector protein that has an important role during infection.

### The effector Cig2 is required for homotypic fusion of CCVs

**Figure 4:**
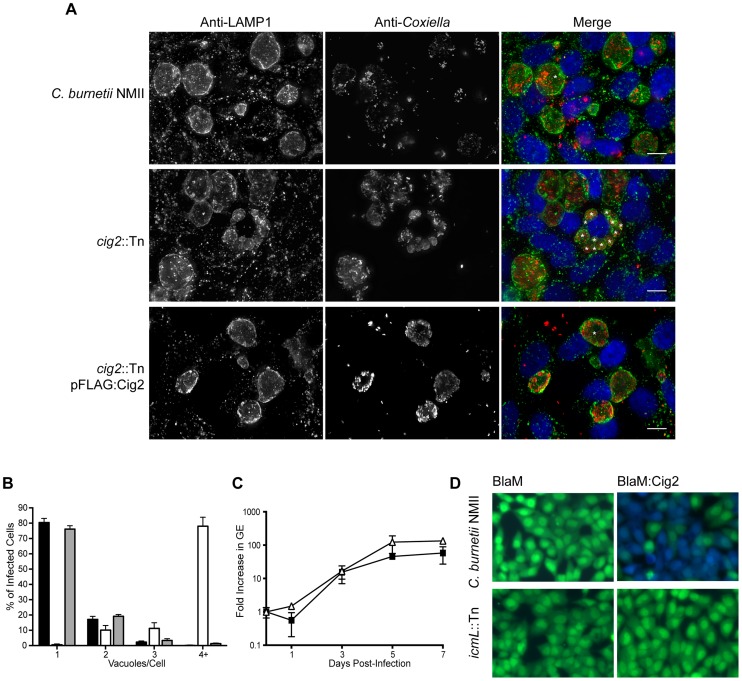
The Dot/Icm effector Cig2 is necessary for homotypic fusion of CCVs. (A) HeLa cells were infected with *C. burnetii* NMII strains at an MOI of 500. Five days post-infection the infections were fixed and stained with anti-*Coxiella* (red), anti-LAMP1 (green) and Hoechst dye (blue). Representative micrographs demonstrate the multiple vacuole phenotype observed for the *cig2*::Tn mutant strain. * indicates the location of individual CCVs within a chosen cell. These CCVs were identified both by LAMP1 staining and phase contrast microscopy. Scale bars represent 10 µm. (B) The average number of vacuoles containing *C. burnetii* NMII (black bars), *cig2*::Tn (white bars) and *cig2*::Tn pFLAG:Cig2 (grey bars) was determined at day 3 post-infection for at least 100 infected cells on duplicate coverslips. Data are the mean ± SD from 3 independent experiments. (C) Replication of *cig2*::Tn (open triangles) and the parental *C. burnetii* NMII strain (black squares) was determined by measuring genome equivalents over a 7 day infection period. (D) Micrographs from translocation assays using *C. burnetii* NMII and the *icmL*::Tn strains producing either BlaM alone (pJB-Cm:BlaM) or BlaM-Cig2 (pJB-Cm:BlaM-Cig2). Fluorescence intensity at 535 nm of uncleaved CCF4-AM is shown in green. Fluorescence intensity of cleaved CCF4-AM at 460 nm generated by the enzymatic activity of BlaM fusion proteins delivered into the host cell cytosol is shown in blue. These images are representative of 3 independent experiments.

A striking phenotype that resulted in the appearance of multiple CCVs in HeLa cells that were infected by *C. burnetii* was observed for 10 independent transposon insertion mutants (Figure 1D). Because individual vacuoles containing replication-competent *C. burnetii* will undergo homotypic fusion inside of an infected cell this phenotype suggests that these mutants were defective in promoting homotypic fusion of the CCV. All of the mutants identified that displayed this multi-vacuolar phenotype had a transposon insertion in the gene encoding the hypothetical protein Cig2 (Cbu0021), which was recently postulated to be an effector because it could be translocated by the *L. pneumophila* Dot/Icm system [45]. The mutants 3-C3 and 1-D12 producing normal CCVs had a transposon insertion in the neighboring gene *cbu0022* and in between *cbu0022* and *cbu0023*, respectively. Thus, it was unlikely that the multi-vacuole phenotype displayed by the *cig2*::Tn insertion mutants was due to a polar effect on expression of downstream genes. When a plasmid encoding the 3×FL-Cig2 protein (pFLAG:Cig2) was introduced into the *cig2*::Tn mutant strain 2-E1 the resulting 2-E1 (pFLAG:Cig2) strain displayed mainly single vacuoles at 72 h post-infection, which was similar to host cells infected with the wild type *C. burnetii* NMII strain under these same conditions (Figure 4). Despite the multi-vacuole phenotype, the *cig2*::Tn mutants formed vacuoles that permit bacterial replication (Figure 4A). Growth curves confirmed that the *cig2*::Tn mutants were not defective for replication in HeLa cells (Figure 4C), which suggests that *cig2* might encode an effector that is required uniquely for processes important for homotypic vacuole fusion. Production of a BlaM-Cig2 protein in *C. burnetii* NMII revealed that Cig2 was translocated during host cell infection and that translocation of BlaM-Cig2 by *C. burnetii* required the Dot/Icm system (Figure 4D). Thus, these data indicate that the Cig2 protein is a translocated effector required for homotypic fusion of the CCV.

### Host autophagy is required for Cig2-dependent homotypic fusion of CCVs

**Figure 5:**
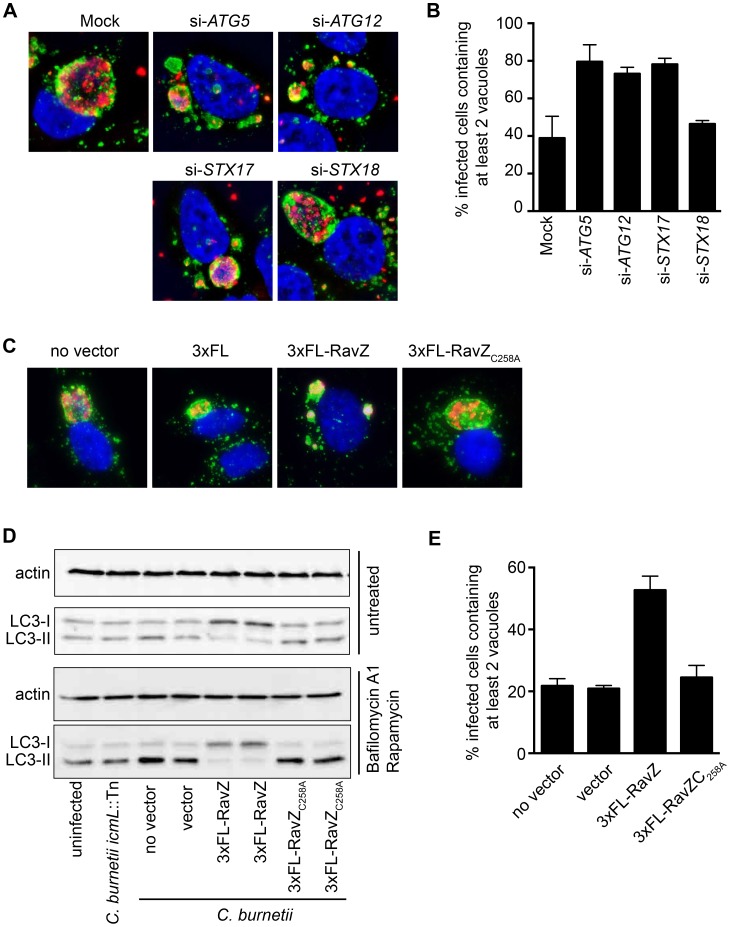
Autophagy is required for homotypic fusion of CCVs. (A) HeLa cells in which the indicated genes were silenced using siRNA were infected with *C. burnetii* and fixed 3 days post-infection before immunostaining with anti-*C. burnetii* (red) and anti-LAMP1 (green) antibodies. DNA was stained with Hoechst (blue). Shown are representative micrographs for mock-transfected cells and cells transfected with siRNA specific to human ATG5, ATG12, STX17, and STX18. (B) Single-cell quantification of *C. burnetii* vacuole counts in ATG5, ATG12, and STX17 siRNA-transfected HeLa cells compared to STX18-transfected cells and mock-transfected cells at 3 days post-infection. The percentages of infected cells with two or more *C. burnetii* vacuoles were counted. Shown is the mean ± SD from 3 independent experiments. (C) HeLa cells infected with *C. burnetii* expressing the *L. pneumophila* effector RavZ from a plasmid (3×FL-RavZ) were assayed 3 days after infection and scored for the multi-vacuolar phenotype. As controls, *C. burnetii* expressing empty vector (3×FL) or a point mutant of RavZ defective in autophagy inhibition (3×FL-RavZ_C258A_) were used. Shown are representative fluorescent images for each *C. burnetii* infection. (D) Immunoblots from uninfected or *C. burnetii*-infected HeLa cell lysates showing bands for non-lipidated (LC3-I) and lipidated (LC3-II) forms of LC3 and actin. Cells were untreated (top images) or treated (bottom images) with bafilomycin A1 and rapamycin for 2 h prior to lysis. Cells were infected with a Dot/Icm-deficient strain of *C. burnetii* (*icmL*::Tn), infected with parental *C. burnetii* NMII (no vector), or *C. burnetii* NMII with vector alone or vectors producing the indicated RavZ proteins. (E) Single-cell quantification of *C. burnetii* vacuole counts in cells at 3 days post-infection. The percentages of infected cells with two or more *C. burnetii* vacuoles were counted. Shown is the mean ± SD from three independent experiments.

Previous studies have revealed a multi-vacuolar phenotype when the host gene encoding Syntaxin-17 (STX17) was silenced in HeLa cells and the STX17-silenced cells were then infected with NMII [8]. The similarity between the phenotype in STX17-silenced cells and the multi-vacuole phenotype observed for the *cig2*::Tn mutant suggested a genetic interaction between Cig2 and STX17. Recent data has shown that STX17 has an essential role in the host process autophagy [57], [58], which would suggest autophagy might be required for homotypic fusion of CCVs. LC3 is a protein that is attached to autophagosomal membranes [59], and is important for autophagosome biogenesis and the selection of intracellular cargo that will be enveloped by autophagy. Consistent with the hypothesis that autophagy may be subverted during *C. burnetii* infection, it has been shown that LC3 is present on the CCV [12]. To determine if autophagy is required for CCV homotypic fusion, we used siRNA to silence the genes encoding the essential autophagy factors ATG5 and ATG12, and vacuole biogenesis was assayed by immunofluorescence microscopy. Compared to mock-transfected cells or cells where the control protein syntaxin-18 (STX18) was silenced, there was a significant increase in the percentage of *C. burnetii*-infected cells having two or more vacuoles per cell when the genes encoding the autophagy factors ATG5, ATG12, or STX17 were silenced (Fig. 5A & B). Thus, a functional host autophagy system is required for Cig2-dependent homotypic fusion of the CCV.

The *L. pneumophila* Dot/Icm effector RavZ is translocated into the host cell during infection and inhibits autophagy by directly uncoupling ATG8 proteins attached to autophagosomal membranes, which includes LC3 [60]. We generated a *C. burnetii* strain that produces 3×FL-RavZ to determine if autophagy is important during the initial stage of infection when the Dot/Icm system is silent or during a later stage of infection when effectors are delivered into host cells. HeLa cells infected with *C. burnetii* producing 3×FL-RavZ had an autophagy defect as determined by the reduction in lipidated LC3-II protein when compared to uninfected cells or cells infected with *C. burnetii* producing the catalytically-inactive 3×FL-RavZ_C258A_ protein (Fig. 5D). Importantly, most of the cells infected with *C. burnetii* producing functional 3×FL-RavZ displayed the multi-vacuolar phenotype defined by the presence of two or more vacuoles containing *C. burnetii*, whereas cells producing the catalytically inactive 3×FL-RavZ_C258A_ protein did not (Fig. 5C & E). Thus, Dot/Icm-mediated delivery of 3×FL-RavZ interfered with homotypic fusion of the CCV by blocking autophagy after bacteria had been transported to a lysosome-derived compartment in the cell, which indicates that Cig2-mediated homotypic fusion of the CCV requires membranes that display lipidated ATG8 proteins.

### The effector Cig2 is important for CCV fusion with autophagosomes

**Figure 6:**
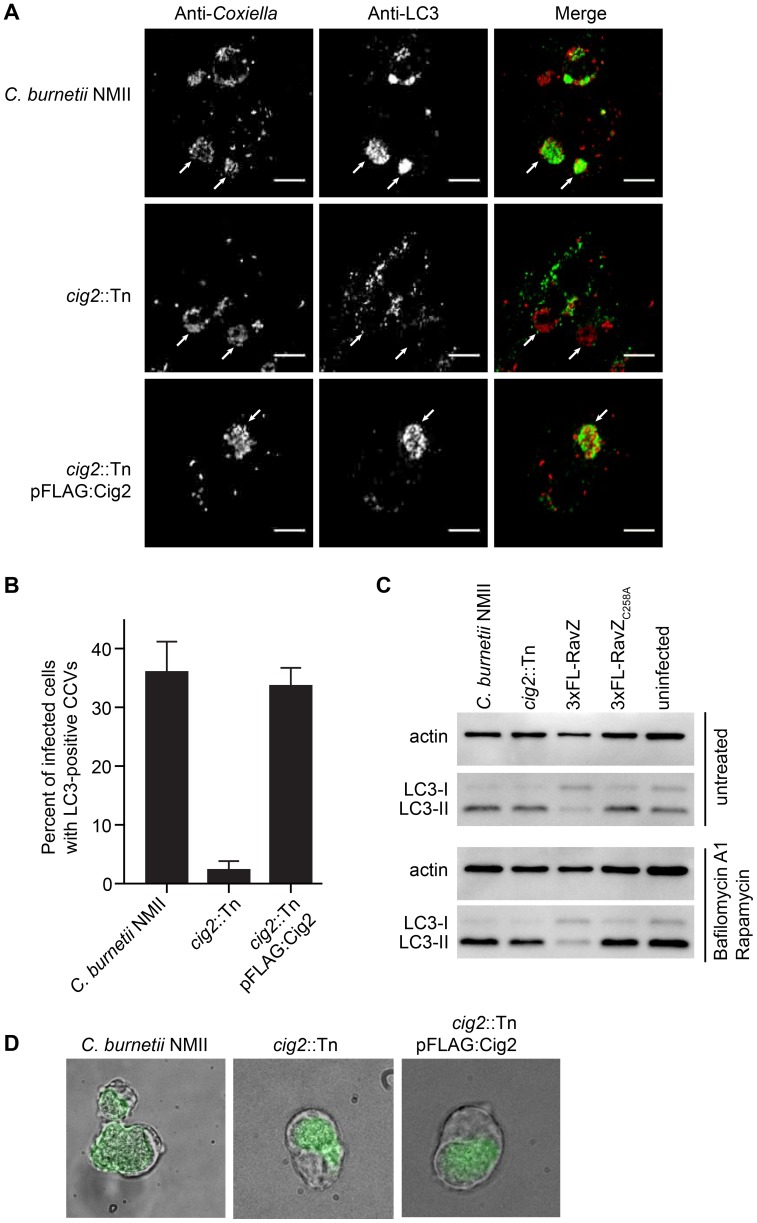
The effector Cig2 is important for CCV fusion with autophagosomes. (A) HeLa cells were infected with *C. burnetii* NMII, *cig2*::Tn or *cig2*::Tn pFLAG:Cig2 for 5 days and stained with anti-LC3 (green) and anti-*Coxiella* (red) antibodies. (B) The association of LC3 with the CCV was quantified by observing 100 infected cells per sample in 3 independent experiments. (C) LC3 and actin immunoblots from HeLa cells infected with parental *C. burnetii* NMII, the *cig2*::Tn mutant, the parental *C. burnetii* NMII producing either RavZ or RavZ_C258A_, or uninfected cells. Cells were untreated (top image), or treated (bottom image) with bafilomycin A1 and rapamycin for 2 h prior to lysis. (D) J774A.1 cells were infected with *C. burnetii* NMII, *cig2*::Tn, or *cig2*::Tn pFLAG:Cig2 for 36 h before loading with 50 µg/ml DQ Green BSA for 16 h. Micrographs show phase contrast images to reveal vacuoles and the green fluorescence that results from DQ Green BSA degradation by proteases in lysosomal compartments.

The finding that defects in host autophagy or loss-of-function mutations in *cig2* both result in a multi-vacuolar phenotype suggested that *C. burnetii* might subvert host autophagy by a Cig2-dependent mechanism. Consistent with this hypothesis we found that the host autophagy protein LC3 was abundant on large vacuoles containing the parental NMII strain, whereas vacuoles containing the isogenic *cig2*::Tn mutant had a severe defect in LC3 accumulation (Figure 6A & B). LC3 accumulation at the CCV was restored when a plasmid encoding 3×FL-Cig2 was introduced into the *cig2*::Tn mutant. To determine if Cig2 may increase autophagy flux in infected cells the autophagy rates were assessed after infection by measuring the accumulation of lipidated LC3-II in cells. There was no appreciable difference in the amounts of lipidated LC3-II detected by immunoblot analysis when cells infected with the *cig2*::Tn mutant strain were compared with cells infected with the parental NMII strain of *C. burnetii* or compared to uninfected cells (Figure 6C). Similar results were observed when autophagy flux was activated through rapamycin treatment of cells and LC3-II levels were stabilized by interfering with lysosome degradation using bafilomycin A1 (Figure 6C). By contrast, a drop in LC3-II levels was measured in cells infected with *C. burnetii* producing 3×FL-RavZ, which results from ability of this effector to deconjugate LC3-II from membranes (Figure 6C). These data indicate that infection by *C. burnetii* does not elevate the basal rate of autophagy and that Cig2 function does not affect autophagy flux during infection.

Lastly, we asked whether the CCV created by the *cig2*::Tn mutant was still accessible to fluid-phase endocytic transport and whether the lumen of the vacuole remained hydrolytic. This question was addressed by pulsing infected macrophages with soluble DQ Green BSA added to the extracellular medium. Endocytic transport and cleavage of DQ Green BSA by lysosomal proteases generates fluorescent peptides that permit visualization of hydrolytic organelles by fluorescence microscopy. Similar to the organelles formed by the parental NMII strain and the *cig2*::Tn mutant complemented with the plasmid producing 3×FLAG-Cig2, the vacuoles containing the Cig2-deficient mutants retained the ability to cleave DQ Green BSA as indicated by the green fluorescence localized to the CCV (Figure 6D), which is consistent with the finding that these vacuoles contain the lysosomal protease Cathepsin D (Figure S1). These data indicate that compared to vacuoles formed by the parental NMII strain, the lumen of the vacuole containing the *cig2*::Tn mutant has a similar capacity to receive endocytic cargo and hydrolyze proteins. Thus, Cig2 function is required to promote fusion of autophagosomes with the initial acidified lysosome-derived vacuole in which *C. burnetii* resides.

## Discussion

Here we employed large-scale transposon mutagenesis to create an arrayed library of 3,237 *C. burnetii* transposon insertion mutants. The *C. burnetii* NMII RSA 493 genome is comprised of a chromosome that is 1,995,275 bp and a 37,393 bp plasmid called QpH1 [30]. The total number of mutants obtained would correlate with at least one insertion for every 628 bp of DNA assuming the transposon we used inserted randomly in the genome. Given that the average size of a *C. burnetii* open reading frame is 849 bp most non-essential genes should be present in the library. Consistent with these calculations, our screen identified insertions in most of the *dot* and *icm* genes predicted to be non-essential for axenic growth of *C. burnetii*. There were, however, also 10 independent insertions isolated in the 2,430 bp gene *cig2*, which is higher than would be predicted given random distribution of the transposon throughout the genome. Thus, we are confident that insertions in most of the genes required for intracellular infection by *C. burnetii* but not for replication in axenic medium were represented in this arrayed library of mutants; however, we acknowledge that there are difficulties in reaching saturation of the genome by transposon mutagenesis that could result in a several genes required for intracellular replication not being present in this library.

We found that loss-of-function mutations in the PmrAB two-component regulatory system abolished intracellular replication of *C. burnetii*, which is consistent with independent data reported in two recent studies [54], [61]. Thus, regulation of the Dot/Icm system and associated effectors by the PmrAB proteins is essential for intracellular replication. Reduced intracellular replication was also observed for *C. burnetii* with insertions in the putative regulatory genes *cbu1761* and *vacB*. The gene *cbu1761* encodes a putative sensor histidine kinase with no apparent cognate response regulator and *vacB* is predicted to encode an exoribonuclease called RNase R. VacB homologs in *Shigella flexneri* and enteroinvasive *Escherichia coli* have been shown to play an important role in host virulence through post-transcriptional positive regulation of plasmid-encoded virulence genes [62], [63]. VacB is thought to mediate this regulatory control through its capacity to process mRNA. This suggests that in addition to PmrAB being required for transcription of genes in the Dot/Icm regulon that there are other virulence-associated circuits controlled by *C. burnetii* regulatory proteins.

Results from this screen provide initial evidence that redox metabolism is important during intracellular replication of *C. burnetii*. A mutant severely impaired for intracellular replication had an insertion in the gene *cbu2072* (Table S1 and Figure S2). The inability to detect translocation of the BlaM-Cbu2072 fusion protein into the host cytosol during *C. burnetii* infection indicates Cbu2072 is unlikely to be an effector protein. Bioinformatic studies predict that the Cbu2072 protein has a molecular weight of 18.2 kDa and limited homology with soluble pyridine nucleotide transhydrogenases (30% identity over 50% of the protein). These enzymes provide an energy-independent means to maintain homeostasis between the two redox factors NAD(H) and NADP(H) [64]. Additional evidence that redox metabolism might be critical during intracellular replication is provided by the mutants 7-G9, 10-B8 and 23-H5 (Table S2), which have independent transposon insertions in the *nadB* gene encoding L-aspartate oxidase. These *C. burnetii nadB* mutants had a moderate intracellular replication defect. NadB catalyzes a step in the quinolinate synthetase complex that generates quinolinic acid from aspartate [65]. Quinolinic acid acts as a precursor for the pyridine nucleotide of NAD. These processes may be specifically important for intracellular replication of *C. burnetii* given the high oxidative stress caused by residing in a lysosome-like organelle.

Several *C. burnetii* mutants were identified in the visual screen because they displayed a filamentous growth phenotype. Disruption of the two-component regulatory system encoded by *cbu2005* and *cbu2006*, *cbu0745*, *mnmA*, *ptsP* and *gidA* resulted in filamentous replication intracellularly. The protein Cbu0745 is predicted to be the *C. burnetii* homolog of ribosome-associated factor Y, and the proteins MnmA and GidA are enzymes involved in tRNA modification. Three independent mutants that displayed a filamentous growth phenotype were found to have insertions in the *gidA* gene, and previous studies indicate that disruption of *gidA* in *Salmonella* also results in bacteria that have defect in cell division resulting in filamentation [66], [67]. This *gidA* mutant phenotype has been attributed to an altered expression of genes responsible for cell division and chromosome segregation [66]. Thus, it is likely that many of the *C. burnetii* mutants that demonstrate filamentation have defects in fundamental cellular processes including translation and chromosome segregation that affect cell division.

Specific Dot/Icm effector proteins critical for CCV biogenesis and intracellular replication of *C. burnetii* were identified in this visual screen. Three other recent studies have reported *C. burnetii* intracellular replication defects resulting from mutations in specific Dot/Icm effectors [46], [48], [54]. By contrast, genetic screens to isolate intracellular replication mutants in *L. pneumophila* identified the Dot/Icm secretion system as being critical for intracellular replication, but did not reveal effector proteins that are essential for intracellular replication. To illustrate this point, it was shown that a *Legionella* strain having five large chromosomal deletions that eliminated the production of 71 different effector proteins could still replicate inside macrophages [68], which provides further evidence that there is extensive functional redundancy built into the *Legionella* effector repertoire and this makes it difficult to identify effectors required for virulence by screening mutants for intracellular replication defects. Thus, the identification of effector mutants with strong intracellular growth phenotypes suggests that there is slightly less functional redundancy in the *C. burnetii* effector repertoire compared to *Legionella*. However, we identified mutants having transposon insertions in genes encoding 16 different effector proteins and were unable to detect any defects in intracellular replication or vacuole morphology for these effector mutants. Thus, it remains likely that there are functionally redundant effectors that modulate some of the host functions required for intracellular replication of *C. burnetii*. Additionally, it is likely that some of the effectors that are encoded by *C. burnetii* play important roles during infection of animals even though these effectors are not required for *C. burnetii* replication in host cells cultured *ex vivo*. Hypothetically, there could be effectors that modulate inflammation by preventing detection of *C. burnetii* by either innate or adaptive immune surveillance that would be predicted to fall into this category.

In our initial attempts at using transposon insertion mutagenesis to identify genes important for intracellular replication we were befuddled by loss-of-function mutations presumably arising spontaneously at a high frequency in *dot* and *icm* genes, which resulted in intracellular growth defects that were not linked to the site of transposon insertion. We optimized the mutagenesis protocol to reduce the probability of phenotypic differences being the result of spontaneous unlinked mutations. By either isolating multiple independent insertions in a gene where all mutants display the same phenotype or by complementing a phenotype by introducing a wild type allele on a plasmid, we demonstrate here that there are distinct phenotypes that are linked to transposon-mediated inactivation of a specific gene. However, it remains possible that some of the mutant phenotypes reported for insertion mutants described in the Supplemental Tables could be due to unlinked mutations and further studies are needed to support this initial analysis. Our data also suggest that unlinked mutations may have complicated results in a recent study where transposon insertions in genes encoding effector proteins were found to affect intracellular replication [46]. Complementation studies were not included in this study, which made it difficult to rule out the possibility that some of the transposon insertion mutants with intracellular growth defects had unlinked mutations that affect Dot/Icm function or the function of some other gene important for infection. For example, it was reported that a *cbu2052* transposon insertion mutant had an intracellular replication defect, however, we obtained two independent mutants with insertions in the *cbu2052* gene and immediately upstream of *cbu2052* (Table S5) and both of these mutants formed CCVs that were indistinguishable from the vacuoles formed by the parental strain of *C. burnetii*. Thus, it is important that transposon insertion phenotypes in *C. burnetii* are validated using either complementation or allelic replacement approaches before important functions are assigned to effector proteins.

Ten intracellular replication mutants isolated in the screen were found to have independent insertions in the *cig57* gene and the intracellular replication defect displayed by a *cig57*::Tn mutant was complemented using a plasmid encoded *cig57* allele. Cig57 is highly conserved among sequenced *C. burnetii* strains, however, database searches did not identify other proteins with homology to Cig57. Thus, we can conclude with high confidence that Cig57 represents a unique effector protein that has an activity that is important for *C. burnetii* intracellular replication.

In addition to identifying mutants defective for intracellular replication, the visual screen revealed that *C. burnetii cig2* mutants display a multi-vacuole phenotype. Whereas infection of a single cell by multiple *C. burnetii* usually leads to formation of a single vacuole due to homotypic fusion of the individual CCVs, the vacuoles containing *cig2* mutants do not display the same propensity to fuse with each other inside the host cell, which results in a single host cell having multiple CCVs that each display LAMP1 and cathepsin D localization at the limiting membrane of the proteolytic lysosome-derived organelle. The *cig2* gene encodes a protein with a predicted molecular weight of 92.9 kDa. The Cig2 protein is encoded in the genomes of all sequenced strains of *C. burnetii*, however, the protein does not have homologs in other organisms and there are no conserved domains that might aid in predicting the biochemical functions of this protein. Our data demonstrate that Cig2 is translocated into host cells during infection by *C. burnetii* using a mechanism that requires the Dot/Icm system. Additionally, it has been shown that Cig2 produced in *Legionella* can be translocated into host cells by the Dot/Icm system [45]. Thus, Cig2 represents a functional Dot/Icm effector protein that modulates vacuole biogenesis.

The multi-vacuolar phenotype displayed by *cig2*::Tn mutants was similar to the multi-vacuolar phenotype displayed after STX17 was silenced and HeLa cells were infected with the parental NMII strain [8]. Why silencing of STX17 would result in a multi-vacuole phenotype was unclear initially, however, recent studies have shown that STX17 function is critical for autophagy in mammalian cells [57], [58]. This suggested that host autophagy was required for homotypic fusion of CCVs. Indeed, our data show that silencing host genes encoding essential component of the autophagy machinery resulted in the multi-vacuole phenotype in *C. burnetii*-infected cells. Additionally, when the LC3-deconjugating effector RavZ was introduced into *C. burnetii*, the RavZ-producing *C. burnetii* were able to disrupt host autophagy and this resulted in a multi-vacuole phenotype. These data provide a clear phenotypic link between the host autophagy system and Cig2 function.

Similar to the unregulated fusion that occurs between pre-existing phagolysosomes and the CCV in infected cells, upregulation of autophagy in mammalian cells generates large autolysosomal organelles as autophagosomes consume lysosomes through rapid fusion [69]. Importantly, independent studies have shown that LC3 associates with the CCV during vacuole biogenesis by an active process mediated by viable *C. burnetii* [12], [13]. Additionally, it has been shown that the presence of LC3 on phagosome membranes will promote rapid fusion with lysosomes by a process known as LC3-associated phagocytosis [70]. Thus, we hypothesized that the reason a *C. burnetii cig2* mutant displays a multi-vacuolar phenotype is because this effector is important for autophagy subversion by *C. burnetii*. Finding that there is defect in the localization of LC3 to vacuoles formed by Cig2-deficient *C. burnetii* supports this hypothesis. Finding that the rates of autophagy were similar following infection of cells with NMII or the isogenic *cig2*::Tn mutant indicates that Cig2 does not stimulate a general upregulation of autophagy flux in the infected cells. This suggests that Cig2 function is required to promote fusion of autophagosomes that are generated at a basal level in the infected cells with the CCV.

Based on these data, we propose a model whereby autophagy subversion by Cig2 is required to constitutively promote the fusion of autophagosomes with the CCV during infection. This would enable Atg8 proteins such as LC3 to be maintained on the CCV membrane and keep the CCV in autolysosomal stage of maturation. We postulate that by locking the CCV in an autolysosomal stage of maturation this vacuole would remain highly fusogenic, and this would promote homotypic fusion and fusion of the CCV with other lysosome-derived organelles in the cell. The result would be formation of a spacious CCV and the fusion of lysosome-derived organelles containing other bacteria or inert particles with the CCV. Determining whether this model is correct will require elucidating the biochemical function of Cig2 and a better understanding of the role autophagy subversion plays in generating the vacuole that *C. burnetii* occupies.

## Materials and Methods

### Bacterial strains and host cell lines

Plaque purified *C. burnetii* Nine Mile phase II (NMII), strain RSA493 clone 4, was axenically grown in liquid ACCM-2 or ACCM-agarose at 37°C in 5% CO_2_ and 2.5% O_2_ as previously described [24], [71]. When appropriate, kanamycin and chloramphenicol were added to ACCM-2 at 300 µg/ml and 3 µg/ml respectively. HeLa 229 cells (CCL-2; ATCC, Manassas, VA) and J774.1 cells were maintained in Dulbecco's Modified Eagle's Media (DMEM) supplemented with 10% heat inactivated fetal bovine serum (FBS) at 37°C in 5% CO_2_.

### Transposon mutagenesis of *C. burnetii* NMII

pKM225 was introduced into stationary phase *C. burnetii* NMII via electroporation at 18 kV, 500 Ω and 25 µF as previously described [28], [71]. Following electroporation, the bacteria were recovered in 20 ml of ACCM-2 for 24 h before being plated on ACCM-agarose plates containing chloramphenicol. After 6 days incubation, single colonies were isolated and resuspended in 1 ml aliquots of ACCM-2 with chloramphenicol in 24 well plates. Following a further 6 days, each 1 ml *C. burnetii* culture was passaged 1∶1000 to provide bacteria for the vacuole formation assay and determination of the transposon insertion site. The remaining culture was pelleted via centrifugation and resuspended in 100 µl of DMEM containing 10% FBS and 10% Dimethyl sulfoxide for storage in 96 well plates.

### Determination of transposon insertion site

The genomic location of the transposon insert sites was determined for transposon mutants with distinct intracellular phenotypes and a wider random selection of recovered mutants. Nested primers within the transposon, facing the transposon-genome junction site, were used to amplify the insertion site either from *C. burnetii* cell lysate or purified genomic DNA. The first round of amplification used primer 1: GGGGGAAACGCCTGGTATC and a pool of random oligonucleotides with a common arm. The product of this PCR was used as a template for the second round of amplification with primer 2: GTCGGGTTTCGCCACCTC and primer ARB2: GGCCAGGCCTGCAGATGATG. The second PCR product was sequenced using primer 3: TCGATTTTTGTGATGCTCGTC. Sequencing results were analyzed using 4Peaks and BLAST programs.

### Vacuole formation assay and immunofluorescence microscopy

1.5×10^4^ HeLa 229 cells were added into 96 well tissue culture plates. The next day monolayers were infected with stationary phase *C. burnetii* transposon mutants at a multiplicity of infection (MOI) of approximately 500 in DMEM with 5% FBS. The infection was allowed to progress for approximately 96 h, with the media changed 24 h after infection. After 96 h, cells were fixed with 4% paraformaldehyde and then blocked and permeablized in blocking buffer (PBS containing 2% BSA and 0.05% saponin). The cells were stained with anti-LAMP1 monoclonal H4A3 (Developmental Studies Hybridoma Bank) and rabbit anti-*C. burnetii* polyclonal antibody in blocking buffer at 1∶1000 and 1∶10000 respectively. Secondary antibodies, Alexa Fluor 488 and 594 (Invitrogen) were used at 1∶3000 also in blocking buffer. During final PBS washes bacterial and host DNA was stained with Hoechst 33342 (Invitrogen). Stained infections were visually inspected for formation of large CCVs and those transposon mutants the exhibited abnormal CCV formation were investigated further. For additional immunofluorescence microscopy HeLa cells were added in 24-well dishes containing 10 mm glass coverslips. At the indicated times post-infection the cells were fixed and stained as above before being mounted on slides using ProLong Gold (Invitrogen). For cathepsin D staining of infected cells, anti-cathepsin D (Novus Biologicals) was used at 1∶50 following fixation and permeablized with ice-cold MeOH and blocking in PBS with 2% BSA. For endogenous LC3 staining HeLa cells were infected for five days before fixing in cold methanol on ice for five minutes. Coverslips were blocked in 2%BSA, and stained with mouse anti-LC3 (NanoTools clone 2G6) and rabbit anti-*Coxiella* antibodies at 1∶200 and 1∶10,000, respectively, in blocking solution. Cells were washed three times in PBS and stained with anti-mouse 488 and anti-rabbit 546 at 1∶2000. Coverslips were washed three times with PBS and mounted on slides using ProLong Gold Antifade reagent (Invitrogen). Images for endogenous LC3 were acquired using an LSM510 confocal microscope equipped with a 100×/1.4 numerical aperture objective lens. Images were analyzed in Image J and Photoshop. For DQ Green BSA experiments, J774A.1 cells were infected with *C. burnetii* NMII, *cig2*::Tn, or *cig2*::Tn pFLAG-Cig2 in 35 mm glass bottom dishes. Cells were incubated in medium containing 50 µg/ml DQ Green BSA at 36 h post-infection and allowed to incubate for a further 16 h. Cells were washed three times with PBS and placed in fresh 5% FBS/DMEM (no phenol red) as described previously [5]. Live images were acquired after one additional hour of incubation with the fresh media. Digital images were acquired with a Nikon Eclipse TE2000-S inverted fluorescence microscope using a 60×/1.4 or 100×/1.4 numerical aperture objective lens and a Photometrics CoolSNAP EZ camera controlled by SlideBook v.5.5 imaging software.

### *C. burnetii* intracellular growth curves

The day before infection, HeLa 229 were plated at a density of 5×10^4^ into 24 well plates with or without 10 mm glass coverslips. Axenically grown stationary phase *C. burnetii* strains were quantified by qPCR using *dotA* specific primers [10] and diluted in DMEM with 5% FBS to an MOI of 50. Following a 4 h infection period, cells were washed once with PBS and incubated with fresh DMEM with 5% FBS. This point was considered Day 0 and a sample was collected to provide the inoculum amount of *C. burnetii*. Infection lysate was collected at the time of infection, 24 (Day 1), 72 (Day 3), 120 (Day 5) and 168 (Day 7) h after this initial time point. Genomic DNA was extracted from these samples using the Illustra Bacteria GenomicPrep Mini Prep Kit (GE Healthcare, Piscataway, NJ) and was used to quantify genomic equivalents by *dotA* specific qPCR. In addition, replicate wells were fixed with 4% paraformaldehyde at Day 3 and Day 5 for subsequent immunofluorescent staining with anti-LAMP1 and anti-*C. burnetii* as described above.

### BlaM translocation assays

Translocation assays were performed as described previously [28], [39]. Genes of interest were cloned into the *Sal*I site of pJB-CAT-BlaM and these constructs were introduced into *C. burnetii* NMII via electroporation. BlaM fusion protein expression of isolated clones was confirmed by western blot with anti-BlaM (1∶1000), (QED Bioscience Inc, San Diego, CA). 2×10^4^ HeLa cells were plated in black clear bottom 96 well trays and, the following day, were infected with stationary phase *C. burnetii* NMII strains at an MOI of 100. The infection was allowed to proceed for 24 h before cells were loaded with the fluorescent substrate CCF4/AM according to the instructions for the LiveBLAzer-FRET B/G Loading Kit (Invitrogen, Carlsbad, CA). Fluorescence, with an excitation of 415 nm and emission at 460 nm and 535 nm, was quantified using a Tecan M1000 plate reader. The ratio of signal at 460 nm to 535 nm (blue:green) was calculated relative to uninfected cells. In addition, cells were visualized by fluorescence microscopy using an inverted Nikon Eclipse TE-2000 S microscope and a 20× objective.

### RNA interference

In 24-well plates, HeLa229 cells were reverse-transfected with small-interfering RNA (siRNA) SMARTpools specific for human *ATG5* (NM_004849), *ATG12* (NM_004707), *STX17* (NM_017919), or *STX18* (NM_016930) using Dharmafect-1 (Thermo Scientific) at a final concentration of 50 nM total siRNA in DMEM with 5% FBS. Transfected cells were incubated overnight, washed, and the adherent cells were subjected to a second round of siRNA transfection at the same concentration. After a two-day incubation, the cells were infected with *C. burnetii* NMII at a MOI of 50. At one day post-infection, the cells were lifted and replated at a lower cell density into a 24-well plate containing 12-mm-diameter glass coverslips and incubated for an additional two days. Cells were processed for immunofluorescence as described above.

### Construction of plasmids encoding RavZ

Primers were designed to amplify *ravZ* or *ravZ*C258A from plasmids pGFP-RavZ or pGFP-RavZ_C258A_ [60] by PCR and to contain extended overhangs specific for sequence- and ligation-independent cloning (SLIC) into the pJB-CAT-3×FLAG destination vector [72]. The following primers show the destination vector sequence underlined and the *ravZ*-specific sequences italicized: RavZ forward, 5′-ATATCGATTACAAGGATGACGATGACAAGGTCGAC *ATGAAAGGCAAGTTAACAGG*-3′ and RavZ reverse, 5′-GGGCGGGGCGTAAAAGCTTGCATGCCTCAGTCGAC *CTATTTTACCTTAATGCCACC*-3′. The resulting vectors pJB-CAT-3×FLAG-RavZ and pJB-CAT-3×FLAG-RavZ_C258A_ encode RavZ proteins that have three tandem copies of the FLAG epitope tag (3×FL) fused to the amino terminus of the protein.

### Infection with *C. burnetii* expressing RavZ

Plasmid DNA (pJB-CAT-3×FLAG-RavZ or pJB-CAT-3×FLAG-RavZ C258A) was electroporated into *C. burnetii* NM II, and chloramphenicol-resistant *C. burnetii* were clonally isolated as described previously [28]. Immunoblots of *C. burnetii* lysates using anti-Flag M2 antibody (Sigma) confirmed that 3×FL-RavZ protein was expressed. *C. burnetii* transformed with pJB-CAT-3×FLAG, pJB-CAT-3×FLAG-RavZ or pJB-CAT-3×FLAG-RavZ C258A were used to infect HeLa cells at a MOI of 50. After 10 h incubation, cells were washed and incubated for three days before either fixation with 4% PFA for immunofluorescence, or lysis for immunoblot analysis.

### LC3 immunoblot analysis

Uninfected or three-day post-infection *C. burnetii*-infected HeLa cells were lysed as described previously for Figure 5 [60]. Lysates were centrifuged and the supernatant separated by SDS-PAGE for immunoblot analysis using an anti-LC3 antibody (Novus) at 1∶3000 and an anti-actin antibody (Sigma) at 1∶5000. For Figure 6, uninfected or five-day post infection *C. burnetii* infected HeLa cells were maintained in 6-well dishes before harvesting with a cell scraper and lysing in buffer containing 2% Triton-X as described in Tanida *et al.*, 2008 [73]. Cells were either left untreated prior to lysis, or were incubated in media containing 200 nM rapamycin and 100 ng/ml bafilomycin A1 2 h prior to lysis.

## Supporting Information

Figure S1 **The CCVs formed by** ***cig57*** **::Tn and** ***cig2*** **::Tn contain the lysosomal protease cathepsin D.** HeLa 229 cells were infected with *C. burnetii* NMII, *cig2*::Tn or *cig57*:Tn at a multiplicity of 50 bacteria to 1 host cell. At 72 h post-infection the samples were fixed and stained with anti-cathepsin D (green), anti-*Coxiella* (red) and Hoechst dye (blue). Representative images demonstrate that cathepsin D is localized to the CCVs formed by all three strains. Scale bars represent 10 µm.
(PDF)

Figure S2 **Cbu1780 is a novel Dot/Icm effector.** The hypothetical proteins Cbu1780 and Cbu2072 were tested for Dot/Icm-dependent translocation using the β-lactamase translocation assay. *C. burnetii* NMII and the *icmL*::Tn mutant expressing BlaM-1780 or BlaM-2072 from a plasmid were used to infect HeLa cells. At 24 h post-infection, CCF4-AM was incorporated into the cells and translocation was assayed through the quantitative measure of fluorescence at 460 nm and 535 nm (A) and visually (B). Translocation positive cells (blue) were only observed for *C. burnetii* NMII expressing BlaM-1780. Results represent the mean 460::535 nm ratio ± standard deviation from three independent experiments and representative images.
(PDF)

Table S1 ***Coxiella burnetii*** **transposon mutants that do not replicate intracellularly.**
(DOCX)

Table S2 ***Coxiella burnetii*** **transposon mutants that display intracellular replication defects.**
(DOCX)

Table S3 ***Coxiella burnetii*** **transposon mutants with a filamentous phenotype.**
(DOCX)

Table S4 **Transposon insertions disrupting** ***cbu0021*** **.**
(DOCX)

Table S5 **Location of transposon insertions in** ***C. burnetii*** **mutants that displayed normal CCV development in the visual screen.**
(DOCX)

## References

[1] Maurin M, Raoult D (1999) Q fever. Clin Microbiol Rev 12: 518–553. 10515901 10.1128/cmr.12.4.518 PMC88923

[2] van der Hoek W, Morroy G, Renders NH, Wever PC, Hermans MH, et al (2012) Epidemic Q fever in humans in the Netherlands. Adv Exp Med Biol 984: 329–364. 22711640 10.1007/978-94-007-4315-1_17

[3] Moos A, Hackstadt T (1987) Comparative virulence of intra- and interstrain lipopolysaccharide variants of *Coxiella burnetii i*n the guinea pig model. Infect Immun 55: 1144–1150. 3570458 10.1128/iai.55.5.1144-1150.1987 PMC260482

[4] Hoover TA, Culp DW, Vodkin MH, Williams JC, Thompson HA (2002) Chromosomal DNA deletions explain phenotypic characteristics of two antigenic variants, phase II and RSA 514 (crazy), of the *Coxiella burnetii* nine mile strain. Infect Immun 70: 6726–6733. 12438347 10.1128/IAI.70.12.6726-6733.2002 PMC132984

[5] Howe D, Shannon JG, Winfree S, Dorward DW, Heinzen RA (2010) *Coxiella burnetii* phase I and II variants replicate with similar kinetics in degradative phagolysosome-like compartments of human macrophages. Infect Immun 78: 3465–3474. 20515926 10.1128/IAI.00406-10 PMC2916283

[6] Baca OG, Akporiaye ET, Aragon AS, Martinez IL, Robles MV, et al (1981) Fate of phase I and phase II *Coxiella burnetii* in several macrophage-like tumor cell lines. Infect Immun 33: 258–266. 7263063 10.1128/iai.33.1.258-266.1981 PMC350684

[7] van Schaik EJ, Chen C, Mertens K, Weber MM, Samuel JE (2013) Molecular pathogenesis of the obligate intracellular bacterium Coxiella burnetii. Nat Rev Microbiol 11: 561–573. 23797173 10.1038/nrmicro3049 PMC4134018

[8] McDonough JA, Newton HJ, Klum S, Swiss R, Agaisse H, et al (2013) Host Pathways Important for *Coxiella burnetii* Infection Revealed by Genome-Wide RNA Interference Screening. MBio 4: e00606–12. 23362322 10.1128/mBio.00606-12 PMC3560531

[9] Hackstadt T, Williams JC (1981) Biochemical stratagem for obligate parasitism of eukaryotic cells by *Coxiella burnetii* . Proc Natl Acad Sci U S A 78: 3240–3244. 6942430 10.1073/pnas.78.5.3240 PMC319537

[10] Coleman SA, Fischer ER, Howe D, Mead DJ, Heinzen RA (2004) Temporal analysis of *Coxiella burnetii* morphological differentiation. J Bacteriol 186: 7344–7352. 15489446 10.1128/JB.186.21.7344-7352.2004 PMC523218

[11] Campoy EM, Zoppino FC, Colombo MI (2011) The early secretory pathway contributes to the growth of the *Coxiella*-replicative niche. Infect Immun 79: 402–413. 20937765 10.1128/IAI.00688-10 PMC3019900

[12] Romano PS, Gutierrez MG, Beron W, Rabinovitch M, Colombo MI (2007) The autophagic pathway is actively modulated by phase II *Coxiella burnetii* to efficiently replicate in the host cell. Cell Microbiol 9: 891–909. 17087732 10.1111/j.1462-5822.2006.00838.x

[13] Gutierrez MG, Vazquez CL, Munafo DB, Zoppino FC, Beron W, et al (2005) Autophagy induction favours the generation and maturation of the *Coxiella*-replicative vacuoles. Cell Microbiol 7: 981–993. 15953030 10.1111/j.1462-5822.2005.00527.x

[14] Heinzen RA, Scidmore MA, Rockey DD, Hackstadt T (1996) Differential interaction with endocytic and exocytic pathways distinguish parasitophorous vacuoles of *Coxiella burnetii* and *Chlamydia trachomatis* . Infect Immun 64: 796–809. 8641784 10.1128/iai.64.3.796-809.1996 PMC173840

[15] Howe D, Heinzen RA (2006) *Coxiella burnetii* inhabits a cholesterol-rich vacuole and influences cellular cholesterol metabolism. Cell Microbiol 8: 496–507. 16469060 10.1111/j.1462-5822.2005.00641.x

[16] Aguilera M, Salinas R, Rosales E, Carminati S, Colombo MI, et al (2009) Actin dynamics and Rho GTPases regulate the size and formation of parasitophorous vacuoles containing *Coxiella burnetii* . Infect Immun 77: 4609–4620. 19635823 10.1128/IAI.00301-09 PMC2747940

[17] Howe D, Melnicakova J, Barak I, Heinzen RA (2003) Fusogenicity of the *Coxiella burnetii* parasitophorous vacuole. Ann N Y Acad Sci 990: 556–562. 12860689 10.1111/j.1749-6632.2003.tb07426.x

[18] Howe D, Melnicakova J, Barak I, Heinzen RA (2003) Maturation of the *Coxiella burnetii* parasitophorous vacuole requires bacterial protein synthesis but not replication. Cell Microbiol 5: 469–480. 12814437 10.1046/j.1462-5822.2003.00293.x

[19] Veras PS, de Chastellier C, Moreau MF, Villiers V, Thibon M, et al (1994) Fusion between large phagocytic vesicles: targeting of yeast and other particulates to phagolysosomes that shelter the bacterium *Coxiella burnetii* or the protozoan *Leishmania amazonensis* in Chinese hamster ovary cells. J Cell Sci 107 Pt 11: 3065–3076. 7699006 10.1242/jcs.107.11.3065

[20] Gomes MS, Paul S, Moreira AL, Appelberg R, Rabinovitch M, et al (1999) Survival of *Mycobacterium avium* and *Mycobacterium tuberculosis* in acidified vacuoles of murine macrophages. Infect Immun 67: 3199–3206. 10377091 10.1128/iai.67.7.3199-3206.1999 PMC116496

[21] Luhrmann A, Roy CR (2007) *Coxiella burnetii i*nhibits activation of host cell apoptosis through a mechanism that involves preventing cytochrome c release from mitochondria. Infect Immun 75: 5282–5289. 17709406 10.1128/IAI.00863-07 PMC2168311

[22] Voth DE, Howe D, Heinzen RA (2007) *Coxiella burnetii* inhibits apoptosis in human THP-1 cells and monkey primary alveolar macrophages. Infect Immun 75: 4263–4271. 17606599 10.1128/IAI.00594-07 PMC1951190

[23] Omsland A, Cockrell DC, Howe D, Fischer ER, Virtaneva K, et al (2009) Host cell-free growth of the Q fever bacterium *Coxiella burnetii* . Proc Natl Acad Sci U S A 106: 4430–4434. 19246385 10.1073/pnas.0812074106 PMC2657411

[24] Omsland A (2012) Axenic growth of *Coxiella burnetii* . Adv Exp Med Biol 984: 215–229. 22711634 10.1007/978-94-007-4315-1_11

[25] Beare PA, Howe D, Cockrell DC, Omsland A, Hansen B, et al (2009) Characterization of a *Coxiella burnetii ftsZ* mutant generated by Himar1 transposon mutagenesis. J Bacteriol 191: 1369–1381. 19114492 10.1128/JB.01580-08 PMC2648191

[26] Beare PA (2012) Genetic manipulation of *Coxiella burnetii* . Adv Exp Med Biol 984: 249–271. 22711636 10.1007/978-94-007-4315-1_13

[27] Beare PA, Larson CL, Gilk SD, Heinzen RA (2012) Two systems for targeted gene deletion in *Coxiella burnetii* . Appl Environ Microbiol 78: 4580–4589. 22522687 10.1128/AEM.00881-12 PMC3370473

[28] Carey KL, Newton HJ, Luhrmann A, Roy CR (2011) The *Coxiella burnetii* Dot/Icm system delivers a unique repertoire of type IV effectors into host cells and is required for intracellular replication. PLoS Pathog 7: e1002056. 21637816 10.1371/journal.ppat.1002056 PMC3102713

[29] Beare PA, Gilk SD, Larson CL, Hill J, Stead CM, et al (2011) Dot/Icm type IVB secretion system requirements for *Coxiella burnetii* growth in human macrophages. MBio 2: e00175–00111. 21862628 10.1128/mBio.00175-11 PMC3163939

[30] Seshadri R, Paulsen IT, Eisen JA, Read TD, Nelson KE, et al (2003) Complete genome sequence of the Q-fever pathogen *Coxiella burnetii* . Proc Natl Acad Sci U S A 100: 5455–5460. 12704232 10.1073/pnas.0931379100 PMC154366

[31] Zamboni DS, McGrath S, Rabinovitch M, Roy CR (2003) *Coxiella burnetii* express type IV secretion system proteins that function similarly to components of the *Legionella pneumophila* Dot/Icm system. Mol Microbiol 49: 965–976. 12890021 10.1046/j.1365-2958.2003.03626.x

[32] Zusman T, Yerushalmi G, Segal G (2003) Functional similarities between the *icm/dot* pathogenesis systems of *Coxiella burnetii* and *Legionella pneumophila* . Infect Immun 71: 3714–3723. 12819052 10.1128/IAI.71.7.3714-3723.2003 PMC161977

[33] Hubber A, Roy CR (2010) Modulation of host cell function by *Legionella pneumophila* type IV effectors. Annu Rev Cell Dev Biol 26: 261–283. 20929312 10.1146/annurev-cellbio-100109-104034

[34] Luo ZQ, Isberg RR (2004) Multiple substrates of the *Legionella pneumophila* Dot/Icm system identified by interbacterial protein transfer. Proc Natl Acad Sci U S A 101: 841–846. 14715899 10.1073/pnas.0304916101 PMC321768

[35] Dorer MS, Kirton D, Bader JS, Isberg RR (2006) RNA interference analysis of *Legionella* in *Drosophila* cells: exploitation of early secretory apparatus dynamics. PLoS Pathog 2: e34. 16652170 10.1371/journal.ppat.0020034 PMC1447669

[36] O'Connor TJ, Boyd D, Dorer MS, Isberg RR (2012) Aggravating genetic interactions allow a solution to redundancy in a bacterial pathogen. Science 338: 1440–1444. 23239729 10.1126/science.1229556 PMC3780440

[37] Roy CR, Berger KH, Isberg RR (1998) *Legionella pneumophila* DotA protein is required for early phagosome trafficking decisions that occur within minutes of bacterial uptake. Mol Microbiol 28: 663–674. 9632267 10.1046/j.1365-2958.1998.00841.x

[38] Nagai H, Cambronne ED, Kagan JC, Amor JC, Kahn RA, et al (2005) A C-terminal translocation signal required for Dot/Icm-dependent delivery of the *Legionella* RalF protein to host cells. Proc Natl Acad Sci U S A 102: 826–831. 15613486 10.1073/pnas.0406239101 PMC545534

[39] Newton HJ, McDonough JA, Roy CR (2013) Effector Protein Translocation by the *Coxiella burnetii* Dot/Icm Type IV Secretion System Requires Endocytic Maturation of the Pathogen-Occupied Vacuole. PLoS One 8: e54566. 23349930 10.1371/journal.pone.0054566 PMC3547880

[40] Pan X, Luhrmann A, Satoh A, Laskowski-Arce MA, Roy CR (2008) Ankyrin repeat proteins comprise a diverse family of bacterial type IV effectors. Science 320: 1651–1654. 18566289 10.1126/science.1158160 PMC2514061

[41] Voth DE, Howe D, Beare PA, Vogel JP, Unsworth N, et al (2009) The *Coxiella burnetii* ankyrin repeat domain-containing protein family is heterogeneous, with C-terminal truncations that influence Dot/Icm-mediated secretion. J Bacteriol 191: 4232–4242. 19411324 10.1128/JB.01656-08 PMC2698476

[42] Chen C, Banga S, Mertens K, Weber MM, Gorbaslieva I, et al (2010) Large-scale identification and translocation of type IV secretion substrates by *Coxiella burnetii* . Proc Natl Acad Sci U S A 107: 21755–21760. 21098666 10.1073/pnas.1010485107 PMC3003115

[43] Luhrmann A, Nogueira CV, Carey KL, Roy CR (2010) Inhibition of pathogen-induced apoptosis by a *Coxiella burnetii* type IV effector protein. Proc Natl Acad Sci U S A 107: 18997–19001. 20944063 10.1073/pnas.1004380107 PMC2973885

[44] Voth DE, Beare PA, Howe D, Sharma UM, Samoilis G, et al (2011) The *Coxiella burnetii* cryptic plasmid is enriched in genes encoding type IV secretion system substrates. J Bacteriol 193: 1493–1503. 21216993 10.1128/JB.01359-10 PMC3067651

[45] Lifshitz Z, Burstein D, Peeri M, Zusman T, Schwartz K, et al (2013) Computational modeling and experimental validation of the *Legionella* and *Coxiella* virulence-related type-IVB secretion signal. Proc Natl Acad Sci U S A 110: E707–715. 23382224 10.1073/pnas.1215278110 PMC3581968

[46] Weber MM, Chen C, Rowin K, Mertens K, Galvan G, et al (2013) Identification of *C. burnetii* type IV secretion substrates required for intracellular replication and *Coxiella*-containing vacuole formation. J Bacteriol 195 17: 3914–24. 23813730 10.1128/JB.00071-13 PMC3754607

[47] Klingenbeck L, Eckart RA, Berens C, Luhrmann A (2012) The *Coxiella burnetii* type IV secretion system substrate CaeB inhibits intrinsic apoptosis at the mitochondrial level. Cell Microbiol doi:10.1111/cmi.12066 [epub ahead of print] 10.1111/cmi.12066 23126667

[48] Larson CL, Beare PA, Howe D, Heinzen RA (2013) *Coxiella burnetii* effector protein subverts clathrin-mediated vesicular trafficking for pathogen vacuole biogenesis. Proc Natl Acad Sci U S A 110: E4770–4779. 24248335 10.1073/pnas.1309195110 PMC3856779

[49] Matthews M, Roy CR (2000) Identification and subcellular localization of the Legionella pneumophila IcmX protein: a factor essential for establishment of a replicative organelle in eukaryotic host cells. Infect Immun 68: 3971–3982. 10858211 10.1128/iai.68.7.3971-3982.2000 PMC101675

[50] Yerushalmi G, Zusman T, Segal G (2005) Additive effect on intracellular growth by *Legionella pneumophila* Icm/Dot proteins containing a lipobox motif. Infect Immun 73: 7578–7587. 16239561 10.1128/IAI.73.11.7578-7587.2005 PMC1273853

[51] Segal G, Shuman HA (1999) Possible origin of the *Legionella pneumophila* virulence genes and their relation to *Coxiella burnetii* . Mol Microbiol 33: 669–670. 10417657 10.1046/j.1365-2958.1999.01511.x

[52] Vogel JP, Andrews HL, Wong SK, Isberg RR (1998) Conjugative transfer by the virulence system of Legionella pneumophila. Science 279: 873–876. 9452389 10.1126/science.279.5352.873

[53] Segal G, Purcell M, Shuman HA (1998) Host cell killing and bacterial conjugation require overlapping sets of genes within a 22-kb region of the Legionella pneumophila genome. Proc Natl Acad Sci U S A 95: 1669–1674. 9465074 10.1073/pnas.95.4.1669 PMC19142

[54] Martinez E, Cantet F, Fava L, Norville I, Bonazzi M (2014) Identification of OmpA, a *Coxiella burnetii* protein involved in host cell invasion, by multi-phenotypic high-content screening. PLoS Pathog 10: e1004013. 24651569 10.1371/journal.ppat.1004013 PMC3961360

[55] Zusman T, Aloni G, Halperin E, Kotzer H, Degtyar E, et al (2007) The response regulator PmrA is a major regulator of the *icm/dot* type IV secretion system in *Legionella pneumophila* and *Coxiella burnetii* . Mol Microbiol 63: 1508–1523. 17302824 10.1111/j.1365-2958.2007.05604.x

[56] Cambronne ED, Roy CR (2007) The Legionella pneumophila IcmSW complex interacts with multiple Dot/Icm effectors to facilitate type IV translocation. PLoS Pathog 3: e188. 18069892 10.1371/journal.ppat.0030188 PMC2134951

[57] Hamasaki M, Furuta N, Matsuda A, Nezu A, Yamamoto A, et al (2013) Autophagosomes form at ER-mitochondria contact sites. Nature 495: 389–393. 23455425 10.1038/nature11910

[58] Itakura E, Kishi-Itakura C, Mizushima N (2012) The Hairpin-type Tail-Anchored SNARE Syntaxin 17 Targets to Autophagosomes for Fusion with Endosomes/Lysosomes. Cell 151: 1256–1269. 23217709 10.1016/j.cell.2012.11.001

[59] Kabeya Y, Mizushima N, Ueno T, Yamamoto A, Kirisako T, et al (2000) LC3, a mammalian homologue of yeast Apg8p, is localized in autophagosome membranes after processing. EMBO J 19: 5720–5728. 11060023 10.1093/emboj/19.21.5720 PMC305793

[60] Choy A, Dancourt J, Mugo B, O'Connor TJ, Isberg RR, et al (2012) The *Legionella* effector RavZ inhibits host autophagy through irreversible Atg8 deconjugation. Science 338: 1072–1076. 23112293 10.1126/science.1227026 PMC3682818

[61] Beare PA, Sandoz KM, Larson CL, Howe D, Kronmiller B, et al (2014) Essential Role for the Response Regulator PmrA in *Coxiella burnetii* Type 4B Secretion and Colonization of Mammalian Host Cells. J Bacteriol 196: 1925–1940. 24610709 10.1128/JB.01532-14 PMC4010987

[62] Tobe T, Sasakawa C, Okada N, Honma Y, Yoshikawa M (1992) *vacB*, a novel chromosomal gene required for expression of virulence genes on the large plasmid of *Shigella flexneri* . J Bacteriol 174: 6359–6367. 1400189 10.1128/jb.174.20.6359-6367.1992 PMC207582

[63] Cheng ZF, Zuo Y, Li Z, Rudd KE, Deutscher MP (1998) The *vacB* gene required for virulence in *Shigella flexneri* and *Escherichia coli* encodes the exoribonuclease RNase R. J Biol Chem 273: 14077–14080. 9603904 10.1074/jbc.273.23.14077

[64] Canonaco F, Hess TA, Heri S, Wang T, Szyperski T, et al (2001) Metabolic flux response to phosphoglucose isomerase knock-out in *Escherichia coli* and impact of overexpression of the soluble transhydrogenase UdhA. FEMS Microbiol Lett 204: 247–252. 11731130 10.1111/j.1574-6968.2001.tb10892.x

[65] Flachmann R, Kunz N, Seifert J, Gutlich M, Wientjes FJ, et al (1988) Molecular biology of pyridine nucleotide biosynthesis in *Escherichia coli*. Cloning and characterization of quinolinate synthesis genes *nadA* and *nadB* . Eur J Biochem 175: 221–228. 2841129 10.1111/j.1432-1033.1988.tb14187.x

[66] Shippy DC, Eakley NM, Bochsler PN, Chopra AK, Fadl AA (2011) Biological and virulence characteristics of *Salmonella enterica* serovar Typhimurium following deletion of glucose-inhibited division (*gidA*) gene. Microb Pathog 50: 303–313. 21320585 10.1016/j.micpath.2011.02.004

[67] von Meyenburg K, Jorgensen BB, Nielsen J, Hansen FG (1982) Promoters of the *atp* operon coding for the membrane-bound ATP synthase of *Escherichia coli* mapped by Tn10 insertion mutations. Mol Gen Genet 188: 240–248. 6185823 10.1007/BF00332682

[68] O'Connor TJ, Adepoju Y, Boyd D, Isberg RR (2011) Minimization of the *Legionella pneumophila* genome reveals chromosomal regions involved in host range expansion. Proc Natl Acad Sci U S A 108: 14733–14740. 21873199 10.1073/pnas.1111678108 PMC3169125

[69] Yu L, McPhee CK, Zheng L, Mardones GA, Rong Y, et al (2010) Termination of autophagy and reformation of lysosomes regulated by mTOR. Nature 465: 942–946. 20526321 10.1038/nature09076 PMC2920749

[70] Sanjuan MA, Dillon CP, Tait SW, Moshiach S, Dorsey F, et al (2007) Toll-like receptor signalling in macrophages links the autophagy pathway to phagocytosis. Nature 450: 1253–1257. 18097414 10.1038/nature06421

[71] Omsland A, Beare PA, Hill J, Cockrell DC, Howe D, et al (2011) Isolation from animal tissue and genetic transformation of *Coxiella burnetii* are facilitated by an improved axenic growth medium. Appl Environ Microbiol 77: 3720–3725. 21478315 10.1128/AEM.02826-10 PMC3127619

[72] Li MZ, Elledge SJ (2012) SLIC: a method for sequence- and ligation-independent cloning. Methods Mol Biol 852: 51–59. 22328425 10.1007/978-1-61779-564-0_5

[73] Tanida I, Ueno T, Kominami E (2008) LC3 and Autophagy. Methods Mol Biol 445: 77–88. 18425443 10.1007/978-1-59745-157-4_4

