# Supplementary figures and images for: A Screen of Coxiella burnetii Mutants Reveals Important Roles for Dot/Icm Effectors and Host Autophagy in Vacuole Biogenesis
Source: PLoS Pathog. 2014 Jul 31;10(7):e1004286. doi: 10.1371/journal.ppat.1004286 (PMC4117601; doi:10.1371/journal.ppat.1004286)

*C. burnetii* NMII

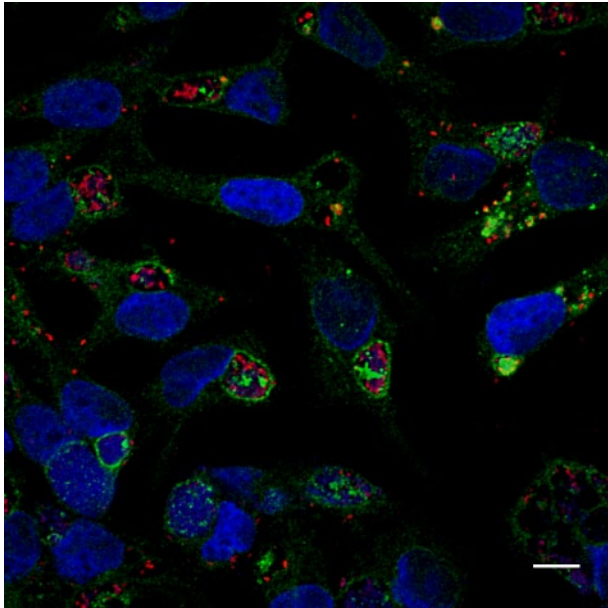

*cig57::Tn*

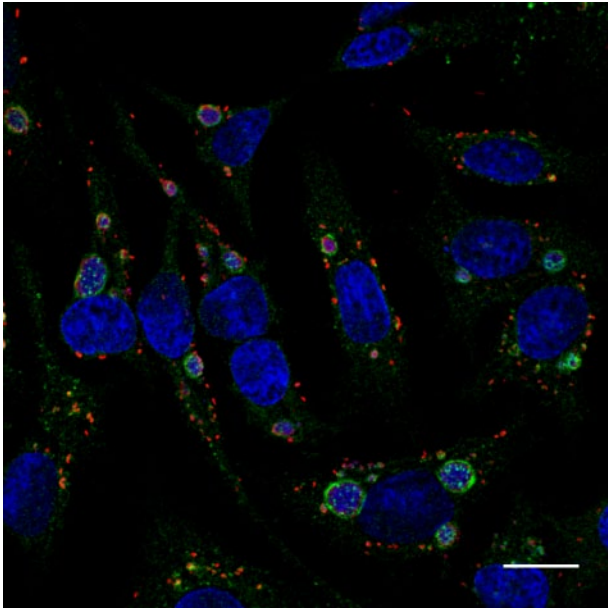

*cig2::Tn*

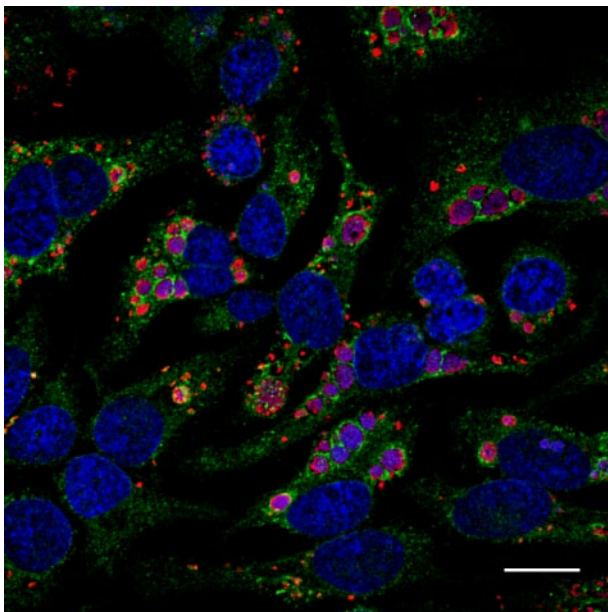

Supplement: Figure S1 — The CCVs formed by cig57 ::Tn and cig2 ::Tn contain the lysosomal protease cathepsin D. HeLa 229 cells were infected with C. burnetii NMII, cig2::Tn or cig57:Tn at a multiplicity of 50 bacteria to 1 host cell. At 72 h post-infection the samples were fixed and stained with anti-cathepsin D (green), anti-Coxiella (red) and Hoechst dye (blue). Representative images demonstrate that cathepsin D is localized to the CCVs formed by all three strains. Scale bars represent 10 µm. (PDF) [file ppat.1004286.s001.pdf]

Supporting Information Figure 2

**A**

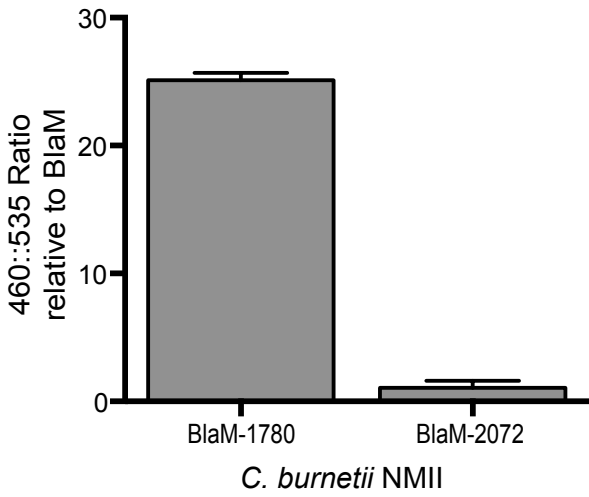

**B**

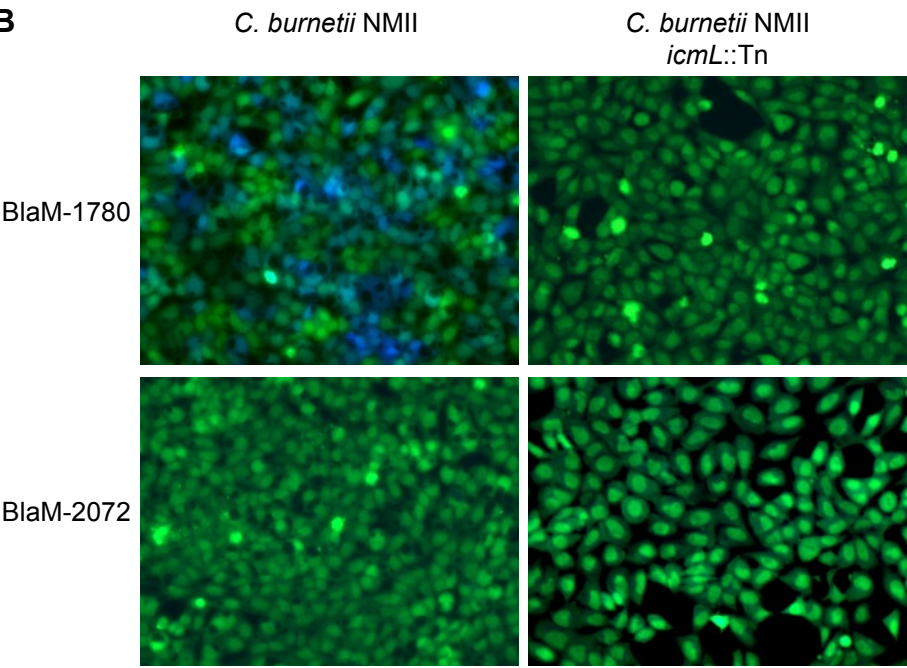

Supplement: Figure S2 — Cbu1780 is a novel Dot/Icm effector. The hypothetical proteins Cbu1780 and Cbu2072 were tested for Dot/Icm-dependent translocation using the β-lactamase translocation assay. C. burnetii NMII and the icmL::Tn mutant expressing BlaM-1780 or BlaM-2072 from a plasmid were used to infect HeLa cells. At 24 h post-infection, CCF4-AM was incorporated into the cells and translocation was assayed through the quantitative measure of fluorescence at 460 nm and 535 nm (A) and visually (B). Translocation positive cells (blue) were only observed for C. burnetii NMII expressing BlaM-1780. Results represent the mean 460::535 nm ratio ± standard deviation from three independent experiments and representative images. (PDF) [file ppat.1004286.s002.pdf]
