# Supplementary material for: A Screen of Coxiella burnetii Mutants Reveals Important Roles for Dot/Icm Effectors and Host Autophagy in Vacuole Biogenesis
Source: PLoS Pathog. 2014 Jul 31;10(7):e1004286. doi: 10.1371/journal.ppat.1004286 (PMC4117601; doi:10.1371/journal.ppat.1004286)
Supplement: Table S1 — Coxiella burnetii transposon mutants that do not replicate intracellularly. (DOCX) [file ppat.1004286.s003.docx]

**Table S1. *Coxiella burnetii* transposon mutants that do not replicate intracellularly.**

| **Disrupted Gene** | **Transposon Insertion Site^1^** | **Mutant** |
| --- | --- | --- |
| **Insertions affecting Dot/Icm function** | | |
| *icmP* | 1574428  1573504  1574438 | 1-D8  8-H10  35-C5 |
| *icmX* | 1591182  1591182  1591041  1591183 | 2-B9  8-C4  23-H9  35-A4 |
| *cbu1651* | 1590505  1590411 | 2-F7  4-C12 |
| *dotA* | 1587001  1588216  1588847  1586564  1587445  1587723  1588340  1587727  1586817  1587228  1588537 | 3-D1  5-D5  9-B1  13-A8  13-B10  13-H5  14-E8  18-G6  27-A12  34-G10  38-E10 |
| *icmK* | 1568992  1568712  1568050  1568462 | 3-D8  4-B7  5-D4  10-A5 |
| *icmL.1* | 1569651  1569201 1569713 | 27-E8  36-B11  40-F11 |
| *icmL.2* | 1570142  1569872  1570145  1570145 | 3-E1  7-D4  12-E9  16-D4 |
| *icmE* | 1565818  1566750  1566811 | 13-B6  20-B4  20-G5 |
| *icmB* | 1561594  1562369  1561694  1560941 | 4-B9  10-B6  15-F8  34-A9 |
| *icmO* | 1572492  1572205  1572458  1572007  1572956  1571801  1572483  1571801  1571800  1572256  1571544 | 5-A9  6-B5  17-C11  18-D9  25-A12  25-F12  27-H9  30-H8  36-D8  37-D10  37-D12 |
| *dotB* | 1583855  1584301  1584063 | 6-B4  12-C2  35-F4 |
| *icmW* | 1589760 | 14-F7 |
| *dotC* | 1583261  1583605 | 18-D2  37-D2 |
| *cbu1634* | 1575374  1575346  1575374  1575389 | 17-D12  25-D10  27-G9  28-H5 |
| *cbu1635* | 1575374 | 24-D12 |
| *icmQ* | 1574545 | 26-B8 |
| *icmJ* | 1562815 | 39-H12 |
| Upstream of *dotA* | 1586654 | 6-F10 |
| Upstream of *icmG* | 1564959 | 27-H3 |
| Upstream of *icmB* | 1562420 | 14-C5 |
| Upstream of *dotB* | 1583607 | 22-B2 |
| **Insertions affecting regulatory genes** | | |
| *pmrA* | 1177661  1177165  1177165 | 4-B8  33-H1  34-B12 |
| *pmrB* | 1178129 | 15-G6 |
| Upstream of *pmrA* | 1176955 | 5-H2 |
| **Insertions affecting ribosomal protein** | | |
| Upstream of *rplE* | 230468 | 23-E8 |
| **Insertions affecting hypothetical proteins** | | |
| *cbu2072* | 1975537 | 8-A8 |
| *cbu1780* | 1708701 | 13-C2 |
