# Supplementary material for: A Screen of Coxiella burnetii Mutants Reveals Important Roles for Dot/Icm Effectors and Host Autophagy in Vacuole Biogenesis
Source: PLoS Pathog. 2014 Jul 31;10(7):e1004286. doi: 10.1371/journal.ppat.1004286 (PMC4117601; doi:10.1371/journal.ppat.1004286)
Supplement: Table S2 — Coxiella burnetii transposon mutants that display intracellular replication defects. (DOCX) [file ppat.1004286.s004.docx]

**Table S2. *Coxiella burnetii* transposon mutants that display moderate intracellular replication defects.**

| **Disrupted Gene** | **Chromosomal**  **Location of Transposon** | **Mutant** |
| --- | --- | --- |
| **Insertions affecting Dot/Icm function** | | |
| *cbu1651* | 1590149 | 6-D12 |
| *icmX* | 1591387 | 12-D2 |
| *icmS* | 1582033 | 25-C3 |
| **Insertions affecting effectors of the Dot/Icm system** | | |
| *cbu1751/cig57/dotDFB5* | 1682347  1681897  1682347  1681773  1682837  1682837  1682201  1681996  1682089  1682201 | 3-H3  11-C4  13-G1  13-H7  20-C4  20-C10  25-E8  31-A12  31-E7  34-E8 |
| *cbu1461/coxCC8* | 417098 | 16-B10 |
| *cbu1754* | 1684955 | 25-D6 |
| **Insertions affecting ribosomal proteins** | | |
| Upstream of *rplL* | 210768 | 3-D3 |
| Upstream of *rplE* | 230467 | 5-A10 |
| *rpsJ* | 225113 | 21-D6 |
| **Insertions affecting proteins involved in metabolic processes** | | |
| *nadB*, L-aspartate oxidase | 95237  95673  95042 | 7-G9  10-B8  23-H5 |
| *cbu0084,* putative phosphoglycerol transferase | 76315  76433 | 6-G8  18-F10 |
| *atpC*, ATP synthase epsilon chain | 1863420 | 13-C1 |
| *dsbD,* thiol:disulfide interchange protein | 1655893 | 20-A12 |
| *cbu1714*, glycine dehydrogenase | 1644255 | 22-E7 |
| *galU*, UTP-glucose-1-phosphate uridylyltransferase | 804904 | 25-D11 |
| **Insertions affecting proteins involved in transport** | | |
| *cbu1896*, putative macrolide efflux protein | 1818464 | 8-A5 |
| *ampG*, muropeptide transporter | 181505 | 8-C10 |
| *cbu0364*, putative phosphate transporter | 330658  330679  330747 | 9-D12  11-E2  27-H5 |
| **Insertions affecting proteins involved in tRNA modification** | | |
| *gidA* | 1841640 | 8-C6 |
| Upstream of *mnmA* | 1090147 | 12-H9 |
| **Insertions affecting putative regulatory proteins** | | |
| *vacB*, ribonuclease R | 1036467 | 29-D9 |
| *cbu1761* | 1693046 | 15-F4 |
| **Insertions affecting hypothetical proteins** | | |
| *cbu1187* | 1130793 | 15-C11 |
| *cbu1119* | 1063253 | 20-H12 |
| *cbu1468* | 1424314 | 25-G5 |
| *cbu0985* | 934576 | 31-F8 |
| *cbu0340* | 308820  308819 | 19-F9  37-B6 |
