# Supplementary material for: A Screen of Coxiella burnetii Mutants Reveals Important Roles for Dot/Icm Effectors and Host Autophagy in Vacuole Biogenesis
Source: PLoS Pathog. 2014 Jul 31;10(7):e1004286. doi: 10.1371/journal.ppat.1004286 (PMC4117601; doi:10.1371/journal.ppat.1004286)
Supplement: Table S3 — Coxiella burnetii transposon mutants with a filamentous phenotype. (DOCX) [file ppat.1004286.s005.docx]

**Table S3. *Coxiella burnetii* transposon mutants with a filamentous phenotype.**

| **Disrupted Gene** | **Chromosomal**  **Location of Transposon** | **Mutant** |
| --- | --- | --- |
| \| *cbu2006* \| \| --- \| | 1912417 | 3-B11 |
| \| *rstB/ cbu2005* \| \| --- \| | 1911800 | 10-A8 |
| \| *ptsP* \| \| --- \| | 1497613 | 3-F2 |
| *gidA* | 1841640  1841678  1840731 | 8-C6  10-E6  32-C11 |
| \| *cbu0745* \| \| --- \| \|  \| | 687490 | 12-G1 |
| *mnmA* | 1090577 | 4-G3 |
| *gcvT* | 1645763 | 31-C5 |
