# Supplementary material for: A Screen of Coxiella burnetii Mutants Reveals Important Roles for Dot/Icm Effectors and Host Autophagy in Vacuole Biogenesis
Source: PLoS Pathog. 2014 Jul 31;10(7):e1004286. doi: 10.1371/journal.ppat.1004286 (PMC4117601; doi:10.1371/journal.ppat.1004286)
Supplement: Table S4 — Transposon insertions disrupting cbu0021 . (DOCX) [file ppat.1004286.s006.docx]

**Table S4. Transposon insertions disrupting *cbu0021*.**

| **Chromosomal**  **Location of Transposon** | **Mutant** |
| --- | --- |
| 18798 | 2-E1 |
| 18991 | 2-H11 |
| 20109 | 8-E5 |
| 20110 | 12-A1 |
| 18798 (same location as 2E1) | 17-C12 |
| 18987 | 26-A3 |
| 19125 | 27-B2 |
| 18988 | 33-A12 |
| 20012 | 37-B5 |
| 20099 | 38-C4 |
