# Supplementary material for: A Screen of Coxiella burnetii Mutants Reveals Important Roles for Dot/Icm Effectors and Host Autophagy in Vacuole Biogenesis
Source: PLoS Pathog. 2014 Jul 31;10(7):e1004286. doi: 10.1371/journal.ppat.1004286 (PMC4117601; doi:10.1371/journal.ppat.1004286)
Supplement: Table S5 — Location of transposon insertions in C. burnetii mutants that displayed normal CCV development in the visual screen. (DOCX) [file ppat.1004286.s007.docx]

**Table S5. Location of transposon insertions in *C. burnetii* mutants that displayed normal CCV development in the visual screen.** Mutants with transposon insertions in genes encoding previously identified Dot/Icm effectors are highlighted in pink.

| **Mutant** | **Chromosomal**  **Location of Transposon** | **Disrupted Gene** |
| --- | --- | --- |
| 1-A1 | 275785 | *htpG* |
| 1-A2 | 53274 | *cbu0056* |
| 1-A3 | 258514 | *rpmB, rpmG* |
| 1-A4 | 199003 | *cbu0215* |
| 1-A5 | 1939731 | *cbu2032* |
| 1-A6 | 246376 | *uvrA* |
| 1-A10 | 1218555 | *cbu1268* |
| 1-A11 | 1087622 | *cbu1146* pseudogene |
| 1-B1 | 1838870 | between *cbu1921* and *trmE* |
| 1-B2 | 36342 of pQpH1 | *cbuA0039* |
| 1-B3 | 50256 | *cbu0054* |
| 1-B4 | 842307 | *cbu0890* |
| 1-B5 | 535122 | *cbu0584* |
| 1-B6 | 1652862 | *cbu1721* |
| 1-B7 | 1577922 | *cbu1638* |
| 1-B8 | 1993301 | *algH* |
| 1-B9 | 50255 | between *enhA.1* and *cbu0054* |
| 1-B10 | 32802 | *fabA* |
| 1-B11 | 438842 | *cbu0498* |
| 1-B12 | 522846 | *cbu0572* |
| 1-C1 | 1605770 | *nlpD* |
| 1-C3 | 1295933 | *cbu1346* |
| 1-C5 | 1679267 | *sspB* |
| 1-C6 | 258918 | *radC*, pseudogene |
| 1-C8 | 777336 | *cbu0827* |
| 1-C11 | 1069503 | between chitinase, pseudogene, and *cbu1127* |
| 1-C12 | 132361 | *ankB*, pseudogene |
| 1-D4 | 1041157 | *cbu1093* |
| 1-D5 | 1936727 | *cbu2029* |
| 1-D7 | 8830 of pQpH1 | *cbua0008b*, pseudogene |
| 1-D9 | 22275 | *csrA-1* |
| 1-D12 | 21818 | between *cbu0022* and *cbu0023* |
| 1-E2 | 272927 | *recG* |
| 1-E3 | 841657 | *dsbA* |
| 1-E4 | 272927 | *recG* |
| 1-E9 | 497071 | *cbu0548* |
| 1-E10 | 49744 | *enhA.1* |
| 1-F1 | 398424 | *corB* |
| 1-F2 | 243150 | *cbu0272* |
| 1-F7 | 602875 | between *dnaZ* and *cbu0658* |
| 1-G8 | 1142971 | *icd* |
| 1-G11 | 1958215 | *cbu2052* |
| 1-H1 | 1163535 | *cbu1217a* |
| 1-H4 | 255248 | *cbu0285* |
| 1-H6 | 1081965 | between *cbu1139* and *cbu1140* |
| 1-H11 | 698736 | *degP.2* |
| 2-A1 | 1110849 | just before start of *cbu1168,* pseudogene |
| 2-A2 | 28668 | *cbu0031* |
| 2-A3 | 9768 of pQpH1 | *cbua0008b* |
| 2-A4 | 998505 | *recA* |
| 2-A6 | 66479 | *ankA* |
| 2-A9 | 1991381 | *cbu2091* |
| 2-B5 | 1931184 | *chiI*, pseudogene |
| 2-B6 | 855850 | *rpmE* |
| 2-B7 | 161623 | *cbu0179* |
| 2-B11 | 340978 | *ampD* |
| 2-C1 | 1495635 | *lgt* |
| 2-C5 | 458007 | *cbu0516a* |
| 2-C11 |  | transposase, multiple locations |
| 2-E3 | 102872 | *cbu0110* |
| 2-E6 | 1896805 | *spa* |
| 2-E7 | 165297 | between *cbu0181a* and rRNA16s |
| 2-F3 | 1316564 | *cbu1367/cbu1368,* pseudogene |
| 2-G4 | 65255 | *ankP*, pseudogene |
| 2-G6 | 34411 | *cbu0038* |
| 2-G9 | 1931805 | *cbu2024* |
| 2-H4 | 662103 | *cbu0715* |
| 2-H5 | 250474 | *cbu0276* |
| 2-H8 | 1797060 | between *cbu1867a* and *CBU1869* |
| 2-H12 | 85666 | *queE* |
| 3-A2 | 182686 | very end of *ampG* |
| 3-A3 | 818188 | *cbu0861*, pseudogene |
| 3-A4 | 1993146 | *pckA* |
| 3-A5 | 450031 | *trpR* |
| 3-A6 | 7887 | *cbu0006A* |
| 3-A7 | 1683964 | *cbu1752* |
| 3-A8 | 698736 | *ubiH* |
| 3-A9 | 1125940 | *cbu1184* |
| 3-A10 | 42049 | between *cbu0043* and *cbu0044* |
| 3-A11 | 1776550 | *cbu1847b* |
| 3-B1 | 1577469 | *cbu1638* |
| 3-B2 | 610585 | *rhlE* |
| 3-B3 | 610585 | *rhlE* |
| 3-B4 | 1922454 | *cbu2015* |
| 3-B7 | 36070 | *prlC* |
| 3-B9 | 1772608 | *cbu1842* |
| 3-B10 | 29304 of pQpH1 | *cbua0032* |
| 3-C1 | 1765381 | *cbu1835* |
| 3-C2 | 1962485 | *cbu2056* |
| 3-C3 | 21470 | *cbu0022* |
| 3-C7 | 1973303 | between *cbu2068* and *cbu2069* |
| 3-C8 | 1640709 | *cbu1710* |
| 3-C11 | 500106 | *pbpA* |
| 3-D2 | 679323 u | *tif* |
| 3-D4 | 1830131 | very end of *com1* |
| 3-D7 |  | transposase, multiple locations |
| 3-D10 | 1979436 | between *hemD* and *cbu2078* |
| 3-D11 | 34736 | *prlC* |
| 3-D12 | 66678 | *ankA* |
| 3-E4 | 1335968 | *rpsB* |
| 3-E5 | 1897522 | *apaH* |
| 3-E6 | 1373750 | *cbu1426*, pseudogene |
| 3-E7 | 9763 of pQpH1 | *cbua0008b* |
| 3-E8 | 1096519 | *CBU1151* |
| 3-E12 | 272962 | *recG* |
| 3-F1 | 1676087 | *cbu1741* |
| 3-F4 | 444944 | *cbu0504* |
| 3-F6 | 1983500 | very end of *hemY* |
| 3-F9 | 1931799 | between *cbu2023* and *cbu2024* |
| 3-F11 | 1766251 | *cbu1836* |
| 3-F12 | 4061 | between *recF* and *gyrB* |
| 3-G1 | 1826075 | *ftsE* |
| 3-G2 | *8780 of pQpH1* | *cbua0008b* |
| 3-G8 | 1983721 | *cbu2082* |
| 3-G9 | 1868973 | *cbu1952* |
| 3-H1 | 352869 | *rpsT* |
| 3-H2 | 49902 | *enhA.1* |
| 3-H5 | 1935082 | between *cbu2027* and *cbu2028* |
| 3-H7 | 89553 | *cbu0095* |
| 3-H11 | 1667975 | *cbu1733* |
| 5-D3 | 26979 | *cbu0029* |
| 5-F9 | 805716 | *cbu0850* |
| 7-D11 | 1280037 | *cbu1334* |
| 7-H5 | 953525 | between *bioA* and *bioB* |
| 7-A12 | 1427791 | between *cbu1472* and *gatC* |
| 10-A1 | 151267 | between *cbu0163* and *cbu0164*, pseudogenes |
| 10-A2 | 1024206 | just before start of *cbu0179* |
| 10-A4 | 280574 | *cbu0313* |
| 10-A12 | 1683027 | *cbu1752* |
| 10-B4 | 1064131 | *cbu1120* |
| 10-B7 | 1864252 | *glmU* |
| 10-B9 | 8048 | just before start of *cbu0006A* |
| 10-B10 | 1127428 | *uvrC* |
| 10-C2 | 10054 | *dacB* |
| 10-C3 | 1830348 | *cbu1911* |
| 10-D5 | 443650 | *cbu0504* |
| 10-E1 | 1208598 | between *ndk* and *nhaP.1* |
| 10-E2 | 308151 | between *cbu0339* and *cbu0040* |
| 10-E8 | 8366 of pQpH1 | *cbua0008a*, pseudogene |
| 10-F1 | 1709804 | between *cbu1780* and *pyk* |
| 10-F11 | 66529 | *ankA* |
| 10-F12 | 697860 | *cbu0754* |
| 10-G12 | 8040 | very start of *cbu0006a* |
| 10-H1 | 1938775 | *ahcY* |
| 10-H4 | 49754 | *enhA.1* |
| 11-A1 | 982843 | *cyoB* |
| 11-A9 | 1081999 | just before start of *cbu1140*, pseudogene |
| 11-B2 | 9405 | *cbu0008a* |
| 11-B3 | 493554 | between *cbu0544* and *lemA* |
| 11-B5 | 1516828 | *cbu1569, coxCC12* |
| 11-C11 | 49461 | between *cbu0051* and *enhA.1* |
| 11-C12 | 385162 | *cbu0431* |
| 11-D8 | 1360102 | *cbu1409* |
| 11-D12 | 193187 | *cbu0207* |
| 11-E9 | 1811943 | *ponA* |
| 11-E10 | 992969 | between tRNA-Ser and *csrA-2* |
| 11-F2 | 1635898 | cbu*1701* |
| 11-F4 | 1971895 | *cbu2067* |
| 11-F9 | 565964 | between *cbu0619* and *lpxB* |
| 11-F11 | 271941 | *recG* |
| 11-F12 | 1701416 | *cbu1770* |
| 11-G4 | 369127 | between tRNA-Lys and *cbu0414*, pseudogene effector (*coxH1)* |
| 11-G6 | 1387006 | between tRNA-Met and *nuoN* |
| 11-G7 | 8820 | *cbu0008* |
| 11-G11 | 429432 | *cbu0488* |
| 11-H1 | 86106 | between *queE* and *clpB* |
| 11-H2 | 1108732 | between *cbu1162* and *cbu1163*, pseudogene |
| 11-H8 | 2758 | *recF* |
| 12-B5 | 1679261 | *sspB* |
| 12-B7 | 1939449 | *cbu2032* |
| 12-B8 | 466918 | *gyrA* |
| 12-C4 | 1992239 | *pckA* |
| 12-C8 | 137342 | *queF* |
| 12-C9 | 1451724 | *cbu1498*, pseudogene |
| 12-D7 | 534419 | *cbu0583* |
| 12-D11 | 698736 | *degP.2* |
| 12-E11 | 1686405 | *cbu1756* |
| 12-E12 | 1941770 | between *cbu2035*, pseudogene, and *cbu2036* |
| 12-F1 | 1686405 | *cbu1756* |
| 12-F5 | 264415 | *xth* |
| 12-F6 | 12637 | *cbu0013* |
| 12-F12 | 1941770 | between *cbu2035*, pseudogene, and *cbu2036* |
| 12-G4 | 57023 | *cbu0062* |
| 12-H3 | 1090170 | between lanthionine synthetase, pseudogene, and *mnmA* |
| 12-H9 | 1090147 | between lanthionine synthetase, pseudogene, and *mnmA* |
| 13-A1 | 1678374 | *cbu1745* |
| 13-A2 | 1678374 | *cbu1745* |
| 13-A5 | 77021 | *cbu0084* |
| 13-A7 | 1678374 | *cbu1745* |
| 13-A12 | 1819250 | *cbu1896a* |
| 13-B2 | 27220 of pQpH1 | *cbu0029a*, pseudogene |
| 13-B3 | 274450 | *cbu0307a* |
| 13-B12 | 357149 | *cbu0393* |
| 13-C3 | 3397 | *recF* |
| 13-C6 |  | transposase, multiple locations |
| 13-C7 | 515722 | *cbu0566* |
| 13-D3 | 1426580 | *mreB* |
| 13-D6 | 58577 | *kdtA* |
| 13-D11 | 1677042 | *gmhA* |
| 13-E2 | 487136 | *smc* |
| 13-E6 | 806226 | between *cbu0850* and *rpsO* |
| 13-E8 | 1578080 | *cbu1638* |
| 13-E10 | 1669620 | *cbu1733* |
| 13-F1 | 1936445 | *cbu2029* |
| 13-F3 | 870283 | *cbu0920* |
| 13-F5 | 521450 | *cbu0571* |
| 13-F10 | 1517306 | *cbu1569* (coxCC12) |
| 13-F11 | 1517306 | *cbu1569* (coxCC12) |
| 13-G3 | 36500 of pQpH1 | *cbua0039a* |
| 13-G4 | 24049 of pQpH1 | *cbua0024* |
| 13-G5 | 10324 of pQpH1 | *cbua0008b* |
| 13-G7 | 1993394 | *algH* |
| 13-G8 | 8363 of pQpH1 | *cbua0008b* |
| 13-G10 | 266832 | *yicC* |
| 13-H1 | 1023981 | *cbu1079* |
| 13-H3 |  | transposase, multiple locations |
| 13-H6 | 654701 | *cbu0705* |
| 13-H9 | 1277657 | *ftsA* |
| 14-A4 | 879480 | *cbu0929* |
| 14-B6 | 1883223 | *cbu1967* |
| 14-B9 | 483574 | between *cbu0539* and *smc* |
| 14-B12 | 429448 | *cbu0488* |
| 14-C6 | 1721437 | *cbu1789* |
| 14-C7 | 36758 of pQpH1 | *cbua0039a* |
| 14-C12 | 1247942 | *cbu1292* |
| 14-D1 | 610807 | *rhlE* |
| 14-D5 | 1978466 | *cbu2076* |
| 14-D6 | 315582 | *cbu0347* |
| 14-D8 | 1326867 | *cbu1376* |
| 14-D12 | 36436 of pQpH1 | *cbua0039a* |
| 14-E3 | 1147712 | *cbu1203* |
| 14-E4 | 1605711 | *cbu1670* |
| 14-E9 | 1683339 | *cbu1752* |
| 14-E10 | 99539 | between *cbu0105*, pseudogene, and *cbu0106* |
| 14-E11 | 1515287 | *ruvC* |
| 14-F1 | 1326095 | *relA* |
| 14-F6 | 1989826 | *cbu2089* |
| 14-F9 | 256078 | *pcnB* |
| 14-F11 | 1828138 | *cbu1907* |
| 14-G1 | 1958784 | just before start of *cbu2052* |
| 14-H6 | 10204 of pQpH1 | *cbua0008b* |
| 14-H9 | 163179 | *cbu0180* |
| 15-F5 | 1683339 | *cbu1752* |
| 15-H9 | 314923 | *xylB* |
| 16-A6 | 7007 | *cbu0006* |
| 16-B11 | 1701416 | *cbu1770* |
| 16-C2 | 689860 | *cbu0749* |
| 16-C4 | 479283 | *cbu0534* |
| 16-C10 | 1973137 | *cbu2068* |
| 16-D3 | 1304794 | *rrmJ* |
| 16-E3 | 1980280 | *cbu2078* |
| 16-F7 | 16242 | *deoC* |
| 16-G9 | 1991126 | *cbu2091* |
| 16-G10 | 1248914 | *cbu1294* |
| 16-H2 | 884336 | *cbu0933* |
| 17-F5 | 683865 | *lon* |
| 17-G11 | 77336 | *cbu0084* |
| 18-A1 | 162544 | *cbu0180* |
| 18-A9 | 332870 | *cbu0368* |
| 18-A10 |  | transposase, multiple locations |
| 18-A11 | 1935656 | *cbu2028* |
| 18-A12 | 1675020 | *cbu1741* |
| 18-B1 | 460975 | *uvrB* |
| 18-B6 | 8780 | *cbu0008a* |
| 18-B10 | 877216 | *mmsA* |
| 18-C1 | 1983723 | *cbu2082* |
| 18-C2 | 1594051 | *rfaE* |
| 18-C5 | 1040749 | *cbu1093* |
| 18-C7 | 281826 | between *cbu0314* and *cbu0315* |
| 18-C11 | 889193 | *cbu0939* |
| 18-D4 | 43471 | *cbu0045* |
| 18-D6 | 479397 | *cbu0534* |
| 18-D8 | 262532 | *cbu0295* |
| 18-E5 | 882039 | between *glpD* and *glpK* |
| 18-E8 |  | transposase, multiple locations |
| 18-E9 | 3957 | between *recF* and *gyrB* |
| 18-E12 | 29599 | *cbu0032* |
| 18-F2 | 1115999 | between *cbu1173* and *cbu1174*, pseudogene |
| 18-G1 | 116558 | *cbu0128/cbu0129* pseudogene |
| 18-G7 | 193187 | *cbu0207* |
| 18-G8 | 1929989 | *cbu2021* |
| 18-G9 | 1904348 | *cbu1998* |
| 18-H6 | 10010 of pQpH1 | *cbua0008b* |
| 18-H8 | 870262 | *cbu0920* |
| 18-H9 | 259272 | *radC,* pseudogene |
| 19-A4 | 1869903 | *cbu1953* |
| 19-A5 | 284765 | *enhA.2* |
| 19-A6 | 246757 | *uvrA* |
| 19-*C5* | 63729 | *ankP*, pseudogene |
| 19-C7 | 1102492 | *trpA* |
| 19-C8 | 67179 | *cbu0073* |
| 19-D5 | 136947 | *cbu0150* |
| 19-D6 | 67176 | *cbu0073* |
| 19-D7 | 27220 of pQpH1 | *cbua0029* |
| 19-D12 | 810719 | *comA* |
| 19-E4 | 881866 | *glpD* |
| 19-E5 | 1043641 | CBU1095; hypothetical exported |
| 19-E6 | 985751 | between *gagA.4* and *cbu1044,* pseudogene |
| 19-E10 | 18092 | *cbu0020* |
| 19-F2 | 490 of pQpH1 | *cbua0001* |
| 19-F5 | 388942 | between *cbu0436* and *cbu0437*, pseudogene |
| 20-A6 | 818797 | *cbu0861* |
| 22-H7 | 523861 | *cbu0573* |
| 23-A6 | 143373 | *cbu1479* |
| 23-A7 | 826319 | *cbu0873* |
| 23-D10 | 36435 of pQpH1 | *cbu0036a*, pseudogene |
| 23-E9 | 385349 | *cbu0432* |
| 23-F5 | 1328215 | *cbu1379* (*coxK2*), pseudogene |
| 23-H6 | 521041 | *cbu0570* |
| 24-C4 | 1579591 | *cbu1639* |
| 25-C11 | 543293 | *cbu0593* |
| 25-F6 | 429029 | *rluC* |
| 25-F11 | 53478 | *cbu0056* |
| 26-G4 | 271707 | *recG* |
| 26-H4 | 471698 | *aroA* |
| 27-E4 | 981991 | *cyoB* |
| 28-B1 | 699855 | *cbu0756* |
| 28-B3 | 1931497 | *chlI*, pseudogene |
| 28-G1 | 1023913 | *cbu1079* |
| 29-A9 | 198089 | *cbu0214* |
| 32-A2 | 1953777 | *metE* |
| 32-A6 | 67750 | *cbu0073* |
| 32-G9 | 47992 | between *cbu0049* and *cbu0050* |
| 39-B12 | 1202529 | *cbu1251*, pseudogene |

^1^  All nucleotide positions refer to locations in the NCBI Reference Sequence NC_002971.3 of the *Coxiella burnetii* RSA 493 chromosome or the NCBI Reference Sequence: NC_004704.1 of the pQpH1 plasmid.
